# Narratives of Backlash? Perceptions of Changing Status Hierarchies in Open-Ended Survey Responses

**DOI:** 10.1177/00104140251392533

**Published:** 2025-11-11

**Authors:** Magdalena Breyer, Tabea Palmtag, Delia Zollinger

**Affiliations:** 1 Institute of Political Science, 27209 University of Basel, Basel, Switzerland; 2 Department of Political Science, 27217 University of Zurich, Zurich, Switzerland

**Keywords:** social status, cultural backlash, progressive politics, open-ended survey questions

## Abstract

It is widely accepted in political science – and remarkably established in public discourse – that status anxieties fuel a far right backlash against progressive politics. This narrative suggests that right-wing conservatives perceive the status of women, racial, or sexual minorities as threatening. Using open-ended survey questions fielded in Germany, we show that women and minorities indeed figure in people’s perceptions of status hierarchies, but in very specific ways: First, overall, people still perceive status as largely socioeconomically determined. Second, sociocultural groups figure in perceptions of who is gaining/losing status, less so in perceptions of the top/bottom of society. Third, more than conservative voters, it is social progressives who mention women and minorities as “winners”. While on race/ethnicity, we find evidence for a backlash, on gender and sexuality we find more evidence for a progressive momentum. This matters for progressive politics today and for how we empirically study status concerns.

## Introduction

The notion that the rise of the far right is rooted in concerns about status, especially among a white male working class, has become one of the most widely perceived and accepted in political science. There is good reason for this, considering a burgeoning literature building on a number of highly influential studies (Burgoon et al., 2019; Cramer, 2016; Engler & Weisstanner, 2021; Gest, 2016; Gidron & Hall, 2017, 2020; Hochschild, 2016; Kurer, 2020; Kurer & Van Staalduinen, 2022; Mutz, 2018; Nachtwey, 2016; Nolan & Weisstanner, 2022). The narrative of a “backlash” against social change was also taken up by the media and has entered public discourse with a remarkable reach and tenacity, spreading from a US and Trump-focused discourse to other countries. Perhaps no other political science theory has been as widely absorbed into public debates in recent years as that according to which conservative “old white men” are threatened by the improved social position of (educated) women, racial, ethnic, or sexual minorities^1^.

In this paper, we use open-ended survey questions from an original, population-representative survey in Germany to study *the extent to which status gains by women or minorities are actually perceived; how these groups feature in people’s perceptions of the social hierarchy; and for whom these aspects of the social order are particularly salient*. The pervasiveness of discussions about shifting hierarchies of gender, race, or sexual orientation suggests that such “sociocultural” factors have become prominent in how people, and especially social conservatives, think about the social rank order. However, we actually know little about which aspects of a backlash narrative are fully articulated in people’s minds, going beyond a diffuse sense of unease and threat triggered by social change (but see Hochschild, 2016; Cramer, 2016 for qualitative work on specific US contexts). Open-ended questions are useful here to capture which societal developments people perceive, welcome, or resent. They do not prime respondents and provide more detailed insights into respondents’ reasoning (Haaland et al., 2024).

We indeed expect the status of women, minorities, as well as graduates to figure in people’s mental maps of society, but in more specific and uneven ways than broad narratives of backlash suggest. We formulate a theoretical framework for thinking about people’s status perceptions in a modern 21st century “knowledge society” (Hall, 2021; Iversen & Soskice, 2019; Kitschelt & Rehm, 2022), along three dimensions: First, we distinguish “socioeconomic” and “sociocultural” status perceptions. We define sociocultural factors as primarily rooted in cultural narratives about the worth and standing of social groups, compared to socioeconomic factors with more direct material and economic foundations. While empirically deeply intertwined with economic inequalities, gender, racial, and sexual hierarchies are thus conceptualized as sociocultural, in line with the literature on the “second dimension” of politics (Hooghe & Marks, 2018; Kitschelt, 1994; Kriesi et al., 2008). Second, we emphasize the importance of *separately* considering people’s perceptions of established hierarchies (a static perspective, i.e. “top versus bottom”) versus their perceptions of ongoing shifts in the social order (a dynamic perspective, i.e. “gaining versus losing” status) (cf. Gest, 2016; Gest et al., 2018). Third, we consider potential heterogeneity in how specific aspects of social hierarchies are perceived across subgroups (e.g., are perceptions of women’s status gains widely shared or unevenly distributed?)

Using quantitative text analyses of our open-ended survey responses and building on recent work e.g. by Rothschild et al., (2019), Zollinger, (2022), or Gidron et al., (2022), we first show that even in times of increased sociocultural polarization, social hierarchies are still perceived largely in directly socioeconomic terms. Second, sociocultural group dynamics (and especially relative status gains of women or minorities) are perceived primarily from a dynamic perspective (losses versus gains), less so when people consider the current state of the social rank order (top versus bottom). These two points taken together strongly suggest that research seeking to study how new forms of sociocultural group conflict matter for politics must tap into how people perceive hierarchies to be *shifting*. For instance, our findings indicate that the widely available “social ladder”-survey item on subjective social status (e.g. Engler & Weisstanner, 2021; Gidron & Hall, 2017) is more likely to capture perceptions of economic hierarchies than new forms of cultural group conflict, due to its static character.

Third, we show that changes in rankings by gender and sexuality are unevenly mentioned across subgroups: more so than social conservatives, it is social progressives (and, e.g., highly educated or younger women) to whom status gains among women or sexual minorities appear disproportionately salient. By contrast, the open-ended responses suggest that shifts in the status of ethnic and racial minorities (“foreigners”, “immigrants”) are perceived to a similar extent by respondents with conservative and progressive attitudes, while varying language clearly reflects the different light in which these subgroups evaluate these perceived developments. Regarding immigration and ethnicity (less so for gender and sexuality), we also see evidence of zero-sum thinking among conservatives, who view “Germans” as losing status while “immigrants” and “foreigners” gain.

In sum, on race and ethnicity, we find evidence of a fully-articulated backlash in how people think about changing social hierarchies; however, on gender and sexuality, we find relatively stronger evidence for progressive momentum. On the one hand, our overall findings hence validate a productive research agenda focused on certain voters’ status concerns: All the main groups that have been theorized as potentially threatening to right-wing, nativist voters appear in our respondents’ descriptions of (changing) social hierarchies. On the other hand, our findings also speak to a growing body of research highlighting the electoral importance of voters who value and care about progressive social change. In the long run, this electoral potential tends to outweigh risks of “backlash” to socially progressive platforms, particularly on the left (Abou-Chadi & Hix, 2021; Breyer, 2024; Abou-Chadi et al., 2025). To this literature, we add evidence that the social position of women and minorities appears relatively more salient to social progressives.

We also corroborate that voters (including social progressives) have not been “distracted” from traditional economic inequalities, as has sometimes been implied by accounts of cultural backlash. Even the socially progressive voters to whom status gains of women or minorities appear disproportionately salient perceive social hierarchies largely in socioeconomic terms. In other words, the open-ended responses show that those people who are most likely to care about advancing gender equality and minority rights are also sensitive to traditional economic inequalities.

## How “Status” has Helped (and Hindered) Our Understanding of Politics in Advanced Democracies

Interest in voters’ status perceptions has increased in part because of the difficulties of explaining emerging political divides purely on the basis of (narrow) material economic circumstances. Initially, this sparked debates about whether economic or cultural factors are primarily responsible for the rise of the far right in opposition to the new left (Manow, 2018; Norris & Inglehart, 2019). Status has gained prominence as a concept that fruitfully enables us to move beyond such a “horse race” perspective and acknowledge that economic and cultural shifts often interact (Bolet, 2021; Engler & Weisstanner, 2021; Gidron & Hall, 2017; Mutz, 2018).

The status concept has helped to make the amalgamation of economic and cultural aspects of structural change explicit. Weber (1978[1922]) famously identified status as a distinctive feature of social stratification, related to but separate from class relations. Embodying broader cultural notions of who is accorded honor, recognition, and respect in society, status (today as in the past) may be rooted in economic position, but also in a more diverse set of characteristics and resources (Breyer, 2025; Gidron & Hall, 2017; Oesch & Vigna, 2022). Recognition, understood as a type of societal valuation structure (Fraser, 2003), and respect are key foundations for status because individuals perceive the social hierarchy in terms of which groups are recognized and respected (Gidron & Hall, 2017; Ridgeway, 2019). Renewed interest in voters’ perceptions of social hierarchies has gone hand in hand with a growing acknowledgment that *relative* (as opposed to absolute) social positions matter for the political divides shaping 21st century politics (Bolet, 2023; Engler & Weisstanner, 2021; Kurer, 2020; Kurer & Van Staalduinen, 2022; Nolan & Weisstanner, 2022; Rooduijn & Burgoon, 2018).

The primary approach in studies focused on how status perceptions advance our understanding of political transformations has been to link voters’ subjective social status to political outcomes. This work typically uses a survey item that asks respondents to place themselves in a social hierarchy, the “social ladder” question (Engler & Weisstanner, 2021; Gidron & Hall, 2017; Kurer, 2020). This research also tends to *infer* “cultural” resentment against improvements in the social position of graduates, women, and minorities from support for the far right among lower-educated, older male workers.

Importantly, though, such self-placements on an abstract social ladder themselves provide no information about how people think about social hierarchies, let alone about changes in the rankings of different groups (for a broader critique of this item, see Anderson, 2025). Beyond qualitative ethnographic work documenting a “cultural backlash” among certain groups disadvantaged by economic and social change (Hochschild, 2016; Cramer, 2016; Gest, 2016, also see Damhuis, 2020; Westheuser, 2021), there is a surprising lack of evidence from representative surveys on how voters think about (changes in) the broader social hierarchy.

Going beyond the focus on subjective social status in recent research, we ask survey respondents to describe various aspects of the social hierarchy as they perceive it, adding to a growing body of work that analyzes open-ended survey questions to study how people perceive society, social conflicts, or group identities (Gidron et al., 2022; Rothschild et al., 2019; Zollinger, 2022). This allows us to directly investigate some of the assumptions made in existing work on status shifts, particularly about social conservatives’ perceptions of how (highly educated) women, racial, ethnic, or sexual minorities rank in the social order. In other words, our interest here is not in voters’ own sense of status but in which aspects of a backlash narrative are fully articulated in people’s mental maps of a changing society.

## Theorizing (Changing) Status Perceptions in 21st-Century Politics

Theoretically, there are good reasons to expect women and minorities to figure in people’s mental maps of society in much more limited and specific ways than the most sweeping accounts of a cultural backlash suggest. To develop this point, we distinguish three dimensions of people’s perceptions of the social order: (1) socioeconomic versus sociocultural group hierarchies (an analytical distinction based on two main dimensions of political conflict in advanced democracies), (2) static versus dynamically changing hierarchies, and (3) aspects of social hierarchies that are homogeneously perceived across society as opposed to heterogeneously across subgroups. Figure 1 visualizes these elements and shows in a simplified manner how we expect those groups most central to “cultural” conflicts – such as women or minorities - to feature in voters’ perceptions of social hierarchies.

**Figure 1.**
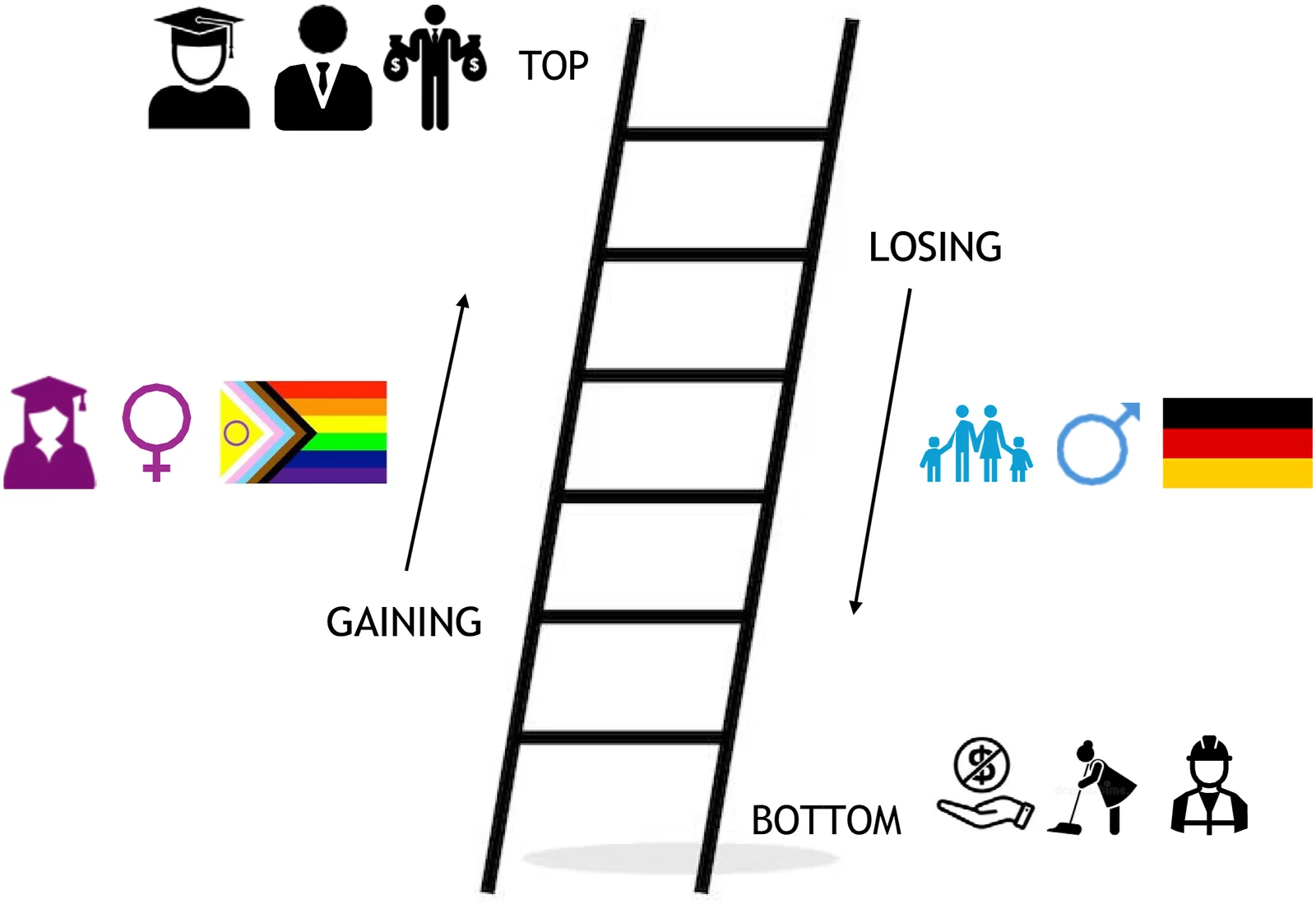
Visualization of theoretical framework - Perceptions of social status (changes).

First, we do *not* expect women, sexual, or racial minorities to be foremost in voters minds when they think about the *top versus bottom* of the social ladder. Although a vast body of work has shown that more “sociocultural” group conflicts associated with a “second” dimension of political competition have become salient and politicized (especially by the far right and the new left, and notably in relation to status; see Kitschelt, 1994; Kriesi et al., 2008; Hooghe & Marks, 2018; Beramendi et al., 2015; Gidron & Hall, 2017; Oesch & Rennwald, 2018; Bornschier et al., 2021), social hierarchies have not been challenged to the point that, say, women would be seen as outranking men in absolute terms. Additionally, gender or (to a lesser degree) ethnic categories cut across vertical class distinctions (cf. Htun, 2004).^2^

In other words, even in times of increased sociocultural conflict or status-based so-called “identity politics”, we can expect traditional “socioeconomic” categories to still be highly relevant for how people think about status in absolute terms. The far-reaching shift from an industrial to a knowledge economy (Hall, 2021; Iversen & Soskice, 2019) has not challenged the fundamentally capitalist nature of our societies, in which economic markers related to wealth, an expensive lifestyle, or highly-remunerated jobs^3^ (see Figure 1) represent a key source and indicator of social valuation (Weber, 1978[1922]; Chan & Goldthorpe, 2007; Savage et al., 2013; Westheuser, 2021). Economic resources have been shown to provide a primary and widely accepted baseline for people’s perception of a hierarchical social order (Harrits & Pedersen, 2018). Broad references to the “rich and the poor” (based on income and wealth) or to “upper and lower” classes (based on economic resources and occupation) are likely to provide the contours for notions of the social hierarchy. If anything, rising inequality in previous decades and the neoliberal turn of the 1980s and 1990s should have further entrenched economic resources and individuals’ success on the market as markers of status (Hall & Lamont, 2013).

To be clear, by analytically distinguishing “socioeconomic” and “sociocultural” groups (shown in black as opposed to color/grayscale in Figure 1), we do not mean to say that the social positions of women or minorities^4^ are unrelated to socioeconomic inequalities (also see Footnote 2). On the contrary, social change is tightly bound up with the shift towards a knowledge society, which has come with an expansion of education, a diversifying class structure, feminization of labor markets, and generally progressive value change (Garritzmann et al., 2022; Gingrich & Häusermann, 2015; Hall, 2021; Iversen & Soskice, 2019; Oesch, 2008). Instead, we base this distinction on the above-cited rich literature that identifies a (first) socioeconomic dimension of political conflict as well as a (second) more sociocultural one.

Our point here is that the group distinctions traditionally associated with “first dimension” state-market conflicts (income, wealth, occupation, and “class” in the traditional sense of the old industrial-era left-right class divide) are still likely to be defining for how voters broadly think about status and status hierarchies, decisively shaping mental images of the top versus bottom ends of social stratification (see Figure 1). The well-documented increase in the salience and politicization of the second dimension suggests that people are increasingly concerned with the social position of groups defined by gender, race, ethnicity, and sexuality. But here, politicization has focused on status *change* (gains/losses that are contested in terms of desirability and deservingness). The same goes for education, which is increasingly associated with second dimension politics (Marks et al., 2022; Kitschelt & Rehm, 2022; Abou-Chadi & Hix, 2021; also see Hooghe et al., 2025 on how educational field relates to this new political divide), even as it is also a long-established traditional source and embodiment of status (Bourdieu, 1984). Figure 1 hence depicts academics as established “at the top” alongside the rich, those in prestigious jobs, etc. – but also shows a (female) graduate as gaining relative status vis-a-vis the traditional male breadwinner, worker, etc.

This brings us directly to our second main point: We expect sociocultural groups to figure in people’s mental maps of society mainly from a *dynamic* perspective. By dynamic, we mean people’s perceptions of how social hierarchies are changing, with some groups gaining and others losing status over time. This point might seem trivial, given that accounts of status anxiety and cultural backlash often do imply shifts in social hierarchies, taking place roughly within the past 50 years (think of Hochschild’s 2016 social escalator analogy). However, existing research is often unclear on this point. Some studies, likely due to data limitations, study survey respondents’ self-placements in static social hierarchies (high, middle, low) while interpreting results based on theories about social change (notably Gidron & Hall, 2017; but see Gest et al., 2018 for an exception). As alluded to above, despite tremendous changes in the awareness, politicization, and steepness of hierarchies of gender, race, or sexuality, these traditional rank orders have seldom been eliminated, let alone reversed. In other words, even if people perceived the social recognition accorded to, say, homosexuals compared to heterosexuals or women as compared to men to have evened out, we would not expect women or LGBTQ+ groups to anchor people’s perceptions of who is at the top of society (also see Figure 1). Importantly, then, static ways of conceptualizing and measuring status perceptions may fail to truly tap into the sociocultural group conflicts that increasingly shape politics.

Note that we do not expect socioeconomic groups to be irrelevant to perceptions of dynamically changing social hierarchies – they may in fact feature prominently.^5^ Although our focus here is on how women, racial, or sexual minorities are most likely to feature, it is to be expected that more straightforwardly socioeconomic factors also figure prominently in perceptions of dynamically changing social hierarchies. This should be the case especially when bound up with new sociocultural conflicts articulated by the far right versus the new left: Politicians and pundits’ talk of liberal “educational elites” opposing a “left behind” working class or “squeezed” white middle class often capture images of relatively changing social positions. Empirically, it is well-established that the far right tends to mobilize historically privileged, fairly well-off, well-protected groups (Bornschier & Kriesi, 2013; Jardina, 2019; Kurer, 2020; Mutz, 2018; Oesch & Rennwald, 2018) while, conversely, the (new) left educated middle class includes groups who are precariously employed or hold underpaid jobs in the social, education, or health sectors (Gingrich & Häusermann, 2015; Häusermann et al., 2015; Abou-Chadi & Hix, 2021; Kitschelt & Rehm, 2022). In other words, just like for women and minorities, absolute social rankings might be murky for some more socioeconomically defined groups while perceived changes in their social standing are salient and politicized.

To summarize our argument so far: where women and minorities *do* loom large in people’s mental maps of society, it should be particularly when people think about ongoing *changes* in who receives social recognition and respect (more so than when people think about current high versus low status). Figure 1 illustrates this simplified expectation.

Third, and keeping our focus on sociocultural groups, we can ask which subgroups are most likely to mention graduates, women, or minorities when thinking about social hierarchies. Put differently, we are interested in variation regarding which elements of the hierarchy illustrated in Figure 1 voters “see” most clearly or saliently. Based on the accounts of cultural backlash serving as our starting point, one might think that socially conservative voters^6^ are especially (resentfully) preoccupied with perceived-to-be improving positions of women, racial, ethnic, or sexual minorities. Certainly, this is an assumption made in much of the recent work on how subjective social status relates to far right support (Engler & Weisstanner, 2021; Gidron & Hall, 2017; Kurer, 2020; Nolan & Weisstanner, 2022). It is also a pattern that could be expected based on ethnographic work documenting socially conservative voters feeling “left behind” when comparing themselves to hitherto disadvantaged social groups (Hochschild, 2016).

However, we also find an alternative expectation plausible: that, in fact, social *progressives* should be particularly likely to perceive and recognize status gains made by women, racial, or ethnic minorities over the past decades. In part, such voters might themselves belong to these groups and be mobilized around political struggles for greater equality. More generally, progressive voters should be particularly concerned with and thus also attuned to gender equality and minority rights, i.e., the left-hand side of the dynamic shifts depicted in Figure 1. From this perspective, socially conservative individuals could be *less* likely to actively acknowledge that women and minorities have gained status, either because these changes are not as salient to them as a “backlash” narrative suggests, because related threat perceptions remain diffuse and weakly articulated, or because they outright negate these groups’ upward trajectory. The latter could be due to political reasons and/or psychological and social motivations to maintain a positive sense of relative status (Lamont, 2000; Shayo, 2009; Tajfel & Turner, 1979). It is possible that, looking at Figure 1, socially conservative voters are relatively more likely to articulate the right-hand side of the depicted status changes: perceived-to-be declining recognition for natives, “normal” people with a traditional lifestyle, etc.^7^

In sum, we expect the social position of (educated) women, ethnic, racial, or sexual minorities to figure in people’s mental maps of the social hierarchy in important but limited and specific ways: We expect them to still be secondary to economic-material resources in how people think about absolute social hierarchies (even in an age of supposed “culture wars”); to feature especially in how people think about dynamically changing social hierarchies rather than the (static) top/bottom of society; and to be unevenly perceived as gaining status across socially progressive versus conservative subgroups.

## Research Design

### Data and Case

To study (shifting) status perceptions, we conducted an original online survey in Germany in 2022. Germany represents a fairly “average” postindustrial economy regarding its economic and sociocultural development in recent years, highlighting the potential of these aspects to feature in status perceptions. Germany’s economic inequality, though not as severe as in Anglo-Saxon countries, has grown (Fuchs-Schündeln et al., 2010), with low intergenerational mobility comparable to the US and UK (Schnitzlein, 2016). Educational inequality is significant, with academic achievement heavily influenced by socioeconomic background, and titles like “Dr.” and “Prof.” are still crucial status indicators, comparable to other European countries like Belgium, France, or Austria. Labor market inequality has risen, marked by a growing low-wage sector (Palier & Thelen, 2010) and restricted welfare access, with public discourse often stigmatizing lower classes. Thus, status perceptions are expected to align closely with socioeconomic groups, similar to other stratified knowledge economies, potentially more so than in Nordic countries relying more on redistribution and less than in the more liberal Anglo-Saxon context.

At the same time, Germany features marked sociocultural inequalities. While its gender pay gap is higher than the EU average (Eurostat, 2024), integration conditions for migrants rank around the Western European mean -but clearly below the Scandinavian countries (Migrant Integration Policy Index, 2020). Likewise, sociocultural political conflicts have intensified, with the early emergence of the Green Party and progressive movements, and a relatively later rise of the radical right AfD compared to other Western European countries (Kriesi et al., 2008; Kurer & Palier, 2019; Oesch & Rennwald, 2018). Public discourses on sociocultural hierarchies have intensified in recent years, especially during the 2015 refugee crisis, highlighting divisions, with parts of society showing solidarity and others rejecting immigrants. Similar debates were visible in many other European countries (and are still ongoing), even though Germans might, for historical reasons, be less prone to mention racial aspects in their status concerns. Similarly, discussions on gender equality, traditional families, and new queer lifestyles are ongoing and increasingly politicized (Mau et al., 2023), with debates on topics like sexuality and trans rights being less polarized than in the US/UK but comparable to other Western European countries. Sociocultural hierarchies could thus also feature saliently in status perceptions. Overall, these perceptions should be comparable to those of other advanced democracies with similar structural and electoral changes.

A total of 3806 respondents took part in the survey and passed attention and speeding checks.^8^ Table A1 in the appendix presents the sample characteristics. The implemented quotas ensure that the sample reflects the population of Germany with regards to education, age, gender, and federate state.^9^

### Measures

Finding out which social groups are meaningful and accessible in individual’s minds when thinking about societal hierarchies is not an easy task for quantitative, survey-based research. Closed-ended questions on where groups like women versus men or high-income versus low-income people stand in society would prime respondents to think about specific aspects of social stratification. In our unprompted framework based on open-ended survey questions, whether and how status groups are described can tell us about the social reality individuals have in mind when thinking about social hierarchies today and over time (see Haaland et al., 2024).

First, to elicit perceptions of *changing social hierarchies*, we asked respondents to describe people who have gained/lost recognition and respect compared to the past. We introduced these questions by stating that “The extent to which groups are recognized and respected in our society changes over time.” We also added information on respondents’ own perceived trajectory based on their previous survey answers: “You yourself previously stated that the social position of people like yourself has rather [decreased/increased/not changed] over the past 30 years.”^10^ Then, the concrete question read: “Now think about the people who have tended to gain recognition compared to the past. How would you describe these people? What kind of characteristics, lifestyles, and opinions do these people have? Just write down what comes to your mind, this is not about right or wrong. Please describe them in a few key words or short sentences.” For perceptions of losing groups, we repeated this wording, but for “people who tend to get less respect compared to before”.

Second, for perceptions of *static hierarchies*, we asked respondents: “How would you describe groups that are currently more on the upper end [in the middle/at the lower end] of society, who receive a lot of [an average amount of/little] recognition?” Again, we encouraged them to name characteristics, lifestyles and opinions of the people they categorize into these three groups. We also presented respondents with their own previous self-placement on the (current) social ladder. In Appendix A2, we show the full question wording, including the way the questions and text fields appeared to respondents. For both changing and static status hierarchies, we emphasize that our focus is on respondents’ descriptions of the perceived change or status quo, rather than their personal preferences or aspirations.^11^

We would like to elaborate on some of our specific decisions regarding question wording: We use the more colloquial terms “respect” and “recognition” (core to the social status definition by Gidron & Hall, 2017, S61) rather than the more abstract term “status”, since we expect it to resonate more with respondents. This is especially true for the German language, where the more common and often interchangeably used words “Respekt” (respect) and “Anerkennung” (recognition) are expected to evoke concrete mental images, whereas “Status” sounds more academic and may be less well understood by some respondents. A concern here might be that “respect” and “recognition” per se trigger more sociocultural group connotations. However, in a pilot wave of the survey, we asked the static question eliciting people’s descriptions of the top/bottom of society without reference to respect/recognition. Appendix A3 shows that, nevertheless, the most frequently mentioned terms across the two survey waves are strikingly consistent, suggesting that people’s responses are not very sensitive to the specific question wording.

Another concern might be that sociocultural categories do not have a “fair” chance against economic ones in a hierarchical setting focused on the “top” and “bottom” of society, given how social worth in our societies is typically monetized and made quantifiable. Besides the fact that this might be substantively true and hence part of what we want to capture, we aimed to level the playing field by *first* asking about perceptions of dynamic shifts, which we expect to be most likely to bring sociocultural groups to mind (and by broadly asking for “characteristics, lifestyles, opinions”). Importantly, we think of “top” and “bottom” not only as inherent in the mental image of hierarchy, but these terms also figure in the wording of the widely used subjective social status item. Using these same terms allows us to cumulatively build on research using this item by studying whether the assumptions it makes about cultural backlash are true.

To classify respondents by their sociocultural ideology, we take the mean support for three items covering typical second-dimension or GAL-TAN topics, namely immigration, EU integration, and gender equality (Häusermann & Kriesi, 2015; Hooghe & Marks, 2018; Oesch & Rennwald, 2018).^12^ In our main analyses, we split this index above the mean to form two groups with socially progressive versus conservative attitudes.

### Text Analysis of Open Questions

Answering open-ended questions about the status hierarchy is a demanding exercise. We therefore first describe how respondents reacted to our open-ended questions before describing our pre-processing and analysis strategy. On average, respondents wrote 10 (10) words when answering the question on who gained (lost) respect and recognition.^13^ The median answer time for questions on perceived status changes was slightly over a minute and a half. The questions about who is at the top, in the middle and at the bottom of society yielded on average 8 to 9 words for each category. Both the average amount of words written and the time spent answering all of the questions indicate that people are able and willing to answer open-ended survey questions related to social status. With roughly 3–4% of answers to either static or dynamic questions comprising less than three characters, this indicates that the large majority of our sample dealt with our questions and made substantial attempts at answering them.

We pre-processed the answers to each open-ended question using the quanteda R package (Benoit et al., 2018), removing answers with fewer than three characters (i.e. non-answers), punctuation, transforming everything to lower case, including frequent and meaningful collocations (combinations of two or three words), and removing stop words (such as “and” or “the”).^14^

When analyzing our open-ended questions, we primarily opt for showing “raw” terms. The great strength of open-ended questions is that we find out about respondents’ mental images of social status hierarchies, based on their own words. Thus, we want to remain close to respondents’ descriptions of status groups. However, we introduce a higher level of abstraction by using a dictionary approach. The dictionary (see Appendix B3) is based on a conceptual distinction between socioeconomic vs. sociocultural aspects of status and group oppositions, in line with our theory. We further consider education as a separate category, since it is of central interest in an emerging knowledge society and widely recognized as linked to material economic outcomes but also to cultural capital. While we started with a list of theoretically defined terms for the dimensions, we then inductively added terms and adapted the dimensions by systematically reviewing frequently mentioned terms.

We opt for a parsimonious dictionary, meaning that we only categorize those terms that are clearly associated with a socioeconomic versus sociocultural dimension of political conflict in the academic literature and broader public debate. First, for the socioeconomic dimension, we include terms referring to occupations/the labor market and to money (encompassing wealth and poverty). Second, the sociocultural dimension includes terms related to gender, race/immigration, and sexuality. While the term “sociocultural” could of course include a range of other aspects, such as disability, age, or religion (also see our theoretical discussion on cultural components of class above), we aim to test the salience of the cultural backlash narrative focusing on its most central categories and catchphrases. Appendix B3 and the presented results based on “raw” responses make our categorizations fully transparent.^15^

## Results

### Predominance of Socioeconomic Status Perceptions

The upper panel of Figure 2 shows the 20 terms occurring most frequently in responses about groups at the top of society, while the lower panel shows terms mentioned most frequently in descriptions of people at the bottom. Socioeconomic terms (black shading) clearly outweigh sociocultural and educational terms (light gray and dark gray shading) for perceptions of both the top and the bottom of society. Terms that we do not attribute clearly to one dimension are shown in white.

**Figure 2.**
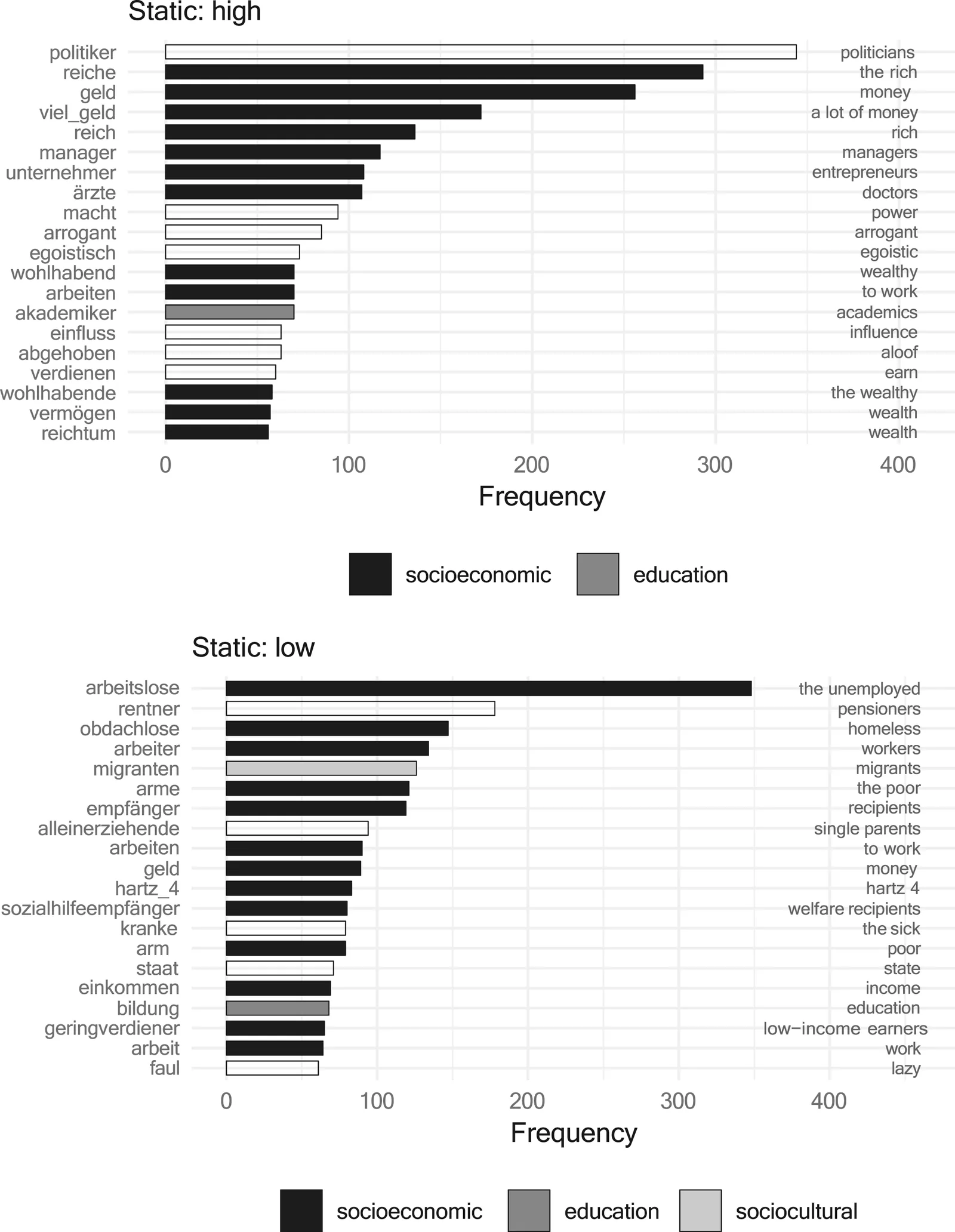
Static status groups - Top 20 absolute frequencies for top and bottom. The frequencies for medium groups are included in Figure C1 in the appendix.

Groups positioned at the top of society are characterized as “the rich”, possessing “a lot of money” or working in elite occupations (“managers” or “doctors”). Another prominent topic relates to political power (“politicians”, “power”), which is neither clearly socioeconomic nor sociocultural in nature. No sociocultural terms appear among the most frequent terms describing the top of society, and “academics” feature as the only educationally defined group. Respondents’ sentiment towards the top of society is indicated by frequently appearing attributes such as “arrogant” or “selfish”. Such negative descriptions of the upper segments of society can be seen as demarcation against “amoral” economic and political elites (Lamont & Molnár, 2002).

Socioeconomic terms also dominate perceptions of those receiving little respect and recognition in society. Amongst a broader set of recurring terms, all referring to people who do not participate in the labor market and depend on social transfers, the “unemployed” are the most frequently mentioned group (bottom panel, Figure 2). References to poverty (“the poor”, “low-income earners”, “income”, “poor”, “money”, as well as “homeless”) appear in low status perceptions and mirror the “wealth” category in descriptions of the top of society.

“Migrants”, are the only sociocultural group to appear within the most frequent terms used to describe the bottom of society. It appears among the top five ranks, which shows that people also acknowledge a more cultural type of disadvantage in the current social hierarchy. However, sociocultural groups are clearly less prevalent in people’s static perceptions of social status overall. In general, people make sense of the current status hierarchy in predominantly socioeconomic terms. An exemplary quote illustrates this close connection respondents see between status and material resources.^16^ To the question of groups at the top of society, a middle-aged woman with medium education answered:

“For me, these are people who earn high profits or salaries. Stock market traders, IT specialists, real estate agents, doctors, lawyers. Money determines recognition... those who earn a lot of it are respected and enjoy high standing in society.”

### Relative Importance of Sociocultural Status Perceptions from a Dynamic Perspective

For *dynamic* status perceptions, we see that mentions of sociocultural groups are relatively more frequent (Figure 3) compared to static perceptions. However, this is only the case for descriptions of status *winners*. The most frequent term here, mentioned almost twice as often as the second most popular word, is “women”. Together with “homosexuals” and “migrants”, these are three important groups commonly associated with sociocultural group conflicts. “Academics” feature in descriptions of status winners, just as they did in perceptions of the top, now accompanied by further references to education (“graduates”, “education”). “Money” and “the rich” still appear frequently but are mentioned less often in comparison to static status terms.

**Figure 3.**
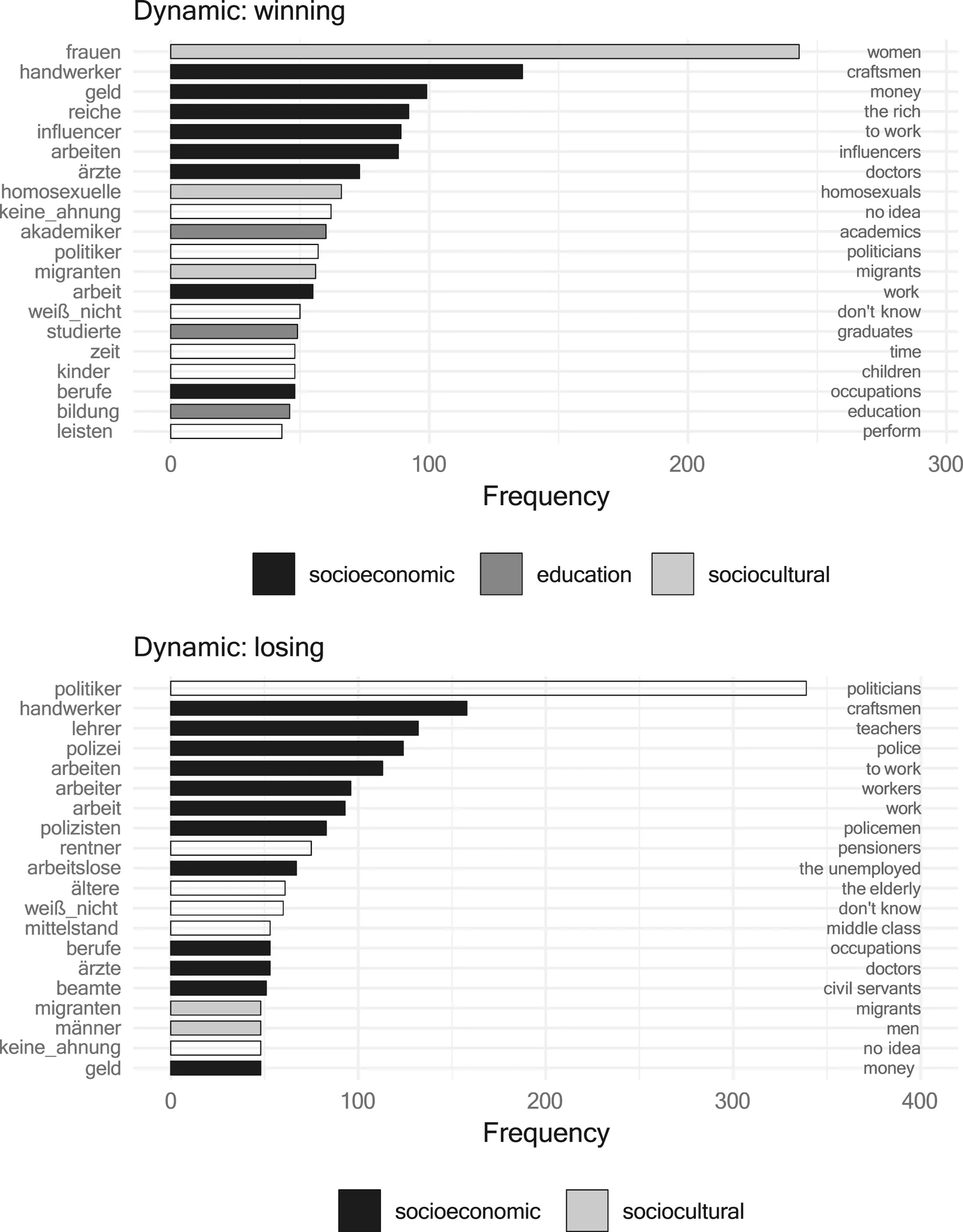
Dynamic status groups - Top 20 absolute frequencies for winners and losers.

While some professions (“doctors” or “craftsmen”) play a role in the description of those who are gaining respect, occupational groups (“craftsmen”, “police”, “teachers”, “civil servants”) are predominant terms for the description of status *losers*.^17^ However, with “migrants” and “men”, two important sociocultural groups also appear here. The fact that men appear among the losing groups and women in descriptions of relative status winners indicates that changing gender relations are to some extent perceived as zero-sum, though mentions of women as winners are a lot more freuquent than those of men as losers. Additionally, what we cannot infer is whether respondents think men rightfully lose some of their status advantage. Interestingly, “craftsmen”, as well as “migrants” appear both when asking people about who *wins* and who *loses* status, indicating somewhat diverging perceptions for changes in occupational and racial hierarchies. The quotes in Table A3 in the appendix illustrate these diverging perceptions. Craftsmen, for example, are perceived to be in high demand by many and therefore are gaining in standing. In contrast, other respondents perceive a general decline in recognition for nonacademic, manual occupations and mention craftsmen as an example. For migrants, some respondents notice different trajectories for highly educated immigrants, who are perceived to be moving up, compared to lower educated migrants perceived to be increasingly scrutinized or discriminated by society.

In general, compared to the static question, “don’t know” answers were more frequent for descriptions of status winners and losers, which could indicate that thinking about status *changes* is more complicated than reporting the perceived status quo.

Our dictionary approach confirms that there is a clear difference between static and dynamic status perceptions regarding the salience of socioeconomic and sociocultural references (see Table 1).^18^ The share of sociocultural groups mentioned in the dynamic questions (11.5%) is more than twice the static share (4.8%). Further, the table corroborates the predominance of socioeconomic categories (47% for static and 38% for dynamic status perceptions). Meanwhile, education-related terms feature similarly for both types of status perceptions (around 9%).

**Table 1.** Salience of Dictionary Dimensions.

Dimension	Question	*N*	%
Socioeconomic	Static	5183	47.2
	Dynamic	2820	38.4
Sociocultural	Static	529	4.8
	Dynamic	847	11.5
Education	Static	940	8.6
	Dynamic	647	8.8
Total	Static	10,987	100.0
	Dynamic	7349	100.0

*Note.* The table displays the percentage of answers per question type that mention a dimension at least once. The dynamic category includes the two questions about winning and losing groups, the static category the three questions about upper, medium and low groups, which is why the Total *N* differs. More detailed results with the full dictionary (see Appendix B3) can be found in Table C1.

### Diverging Perceptions of Status Shifts Between Social Progressives and Social Conservatives

To understand how status shifts matter for electoral conflicts in the 21st century, we contrast responses by individuals with progressive versus conservative attitudes on second dimension issues. We present keyness scores, which identify the most *characteristic* terms mentioned by these subgroups. The following two plots present the twenty terms mentioned with greatest relative frequency by socially progressive and conservative respondents respectively. The more the chi-squared values deviate from zero, the more characteristic a term is for an attitudinal group. Importantly, words mentioned with similar frequency by both groups will not appear in these plots, even if they are important in absolute terms.

For perceptions of groups that are winning status, we find a notable divergence by attitudinal subgroups for the salience of sociocultural groups (Figure 4). Terms characteristically used by individuals with conservative attitudes are shown at the bottom, while words used by progressives are at the top. Rather than sociocultural groups (e.g. women or LGBTQ+), terms like “politicians” and “the rich” are most distinctive for right-wing conservative descriptions of status winners. However, “immigrants”, “foreigners” and “asylum seekers” also set social conservatives’ descriptions of status winners apart.

**Figure 4.**
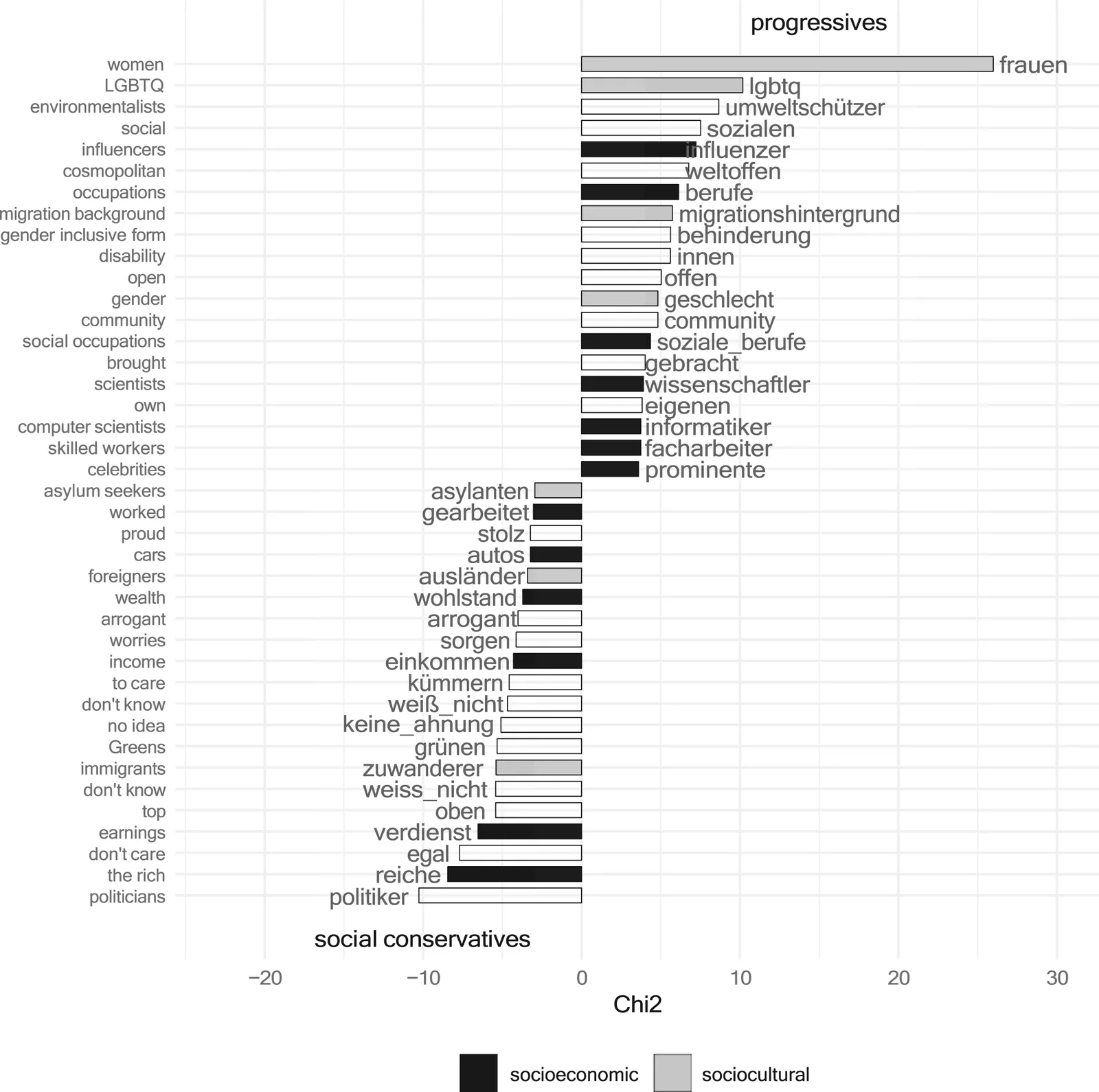
Keyness scores - Subgroup comparison of groups mentioned as gaining status. The plot displays the 20 most characteristic words used to describe winning groups, by progressive (top) vs. conservative attitudes (bottom). The more extreme the Chi-squared statistic (= keyness score) on the *x*-axis is, the more characteristic (or comparatively frequent) a term is for one group compared to the other.

In contrast to social conservatives, progressives perceive status gains by “women” (gender), “LGBTQ” (sexuality) people, and those with a “migration background” (race) with greater relative frequency, touching on dimensions central to the backlash narrative. In other words, recognizing improvements in the social position of women and LGBTQ+ people distinguishes progressives’ responses from those of social conservatives.^19^ Terms surrounding migration and race,^20^ on the other hand, appear as distinctive for both groups, suggesting that this is the more overtly contested dimensions of changing status hierarchies; this aspect features in the keyness statistics because both groups use different language to refer to immigrants (“migration background” among progressives versus “foreigners” or “immigrants” among social conservatives).

For perceptions of status loss, social conservatives most distinctively refer to “Germans” (Figure 5). The conjunction with “country”, “honest people” and “citizens” (unclassified but topically related to descriptions of the majority population) indicates that a sense of status decline for native (white) Germans is highly characteristic for social conservatives. It is important to note that mentioning a unified “German people” as a losing group carries particular weight given Germany’s Nazi past (Marcuse, 2008). While such nationalist sentiments are culturally more frowned upon than in other countries, our open questions register an ongoing norm shift with regards to nationalist identities and narratives, underlining that people feel free to express their status concerns (despite potentially interfering social norms, see Miller-Idriss, 2009).

**Figure 5.**
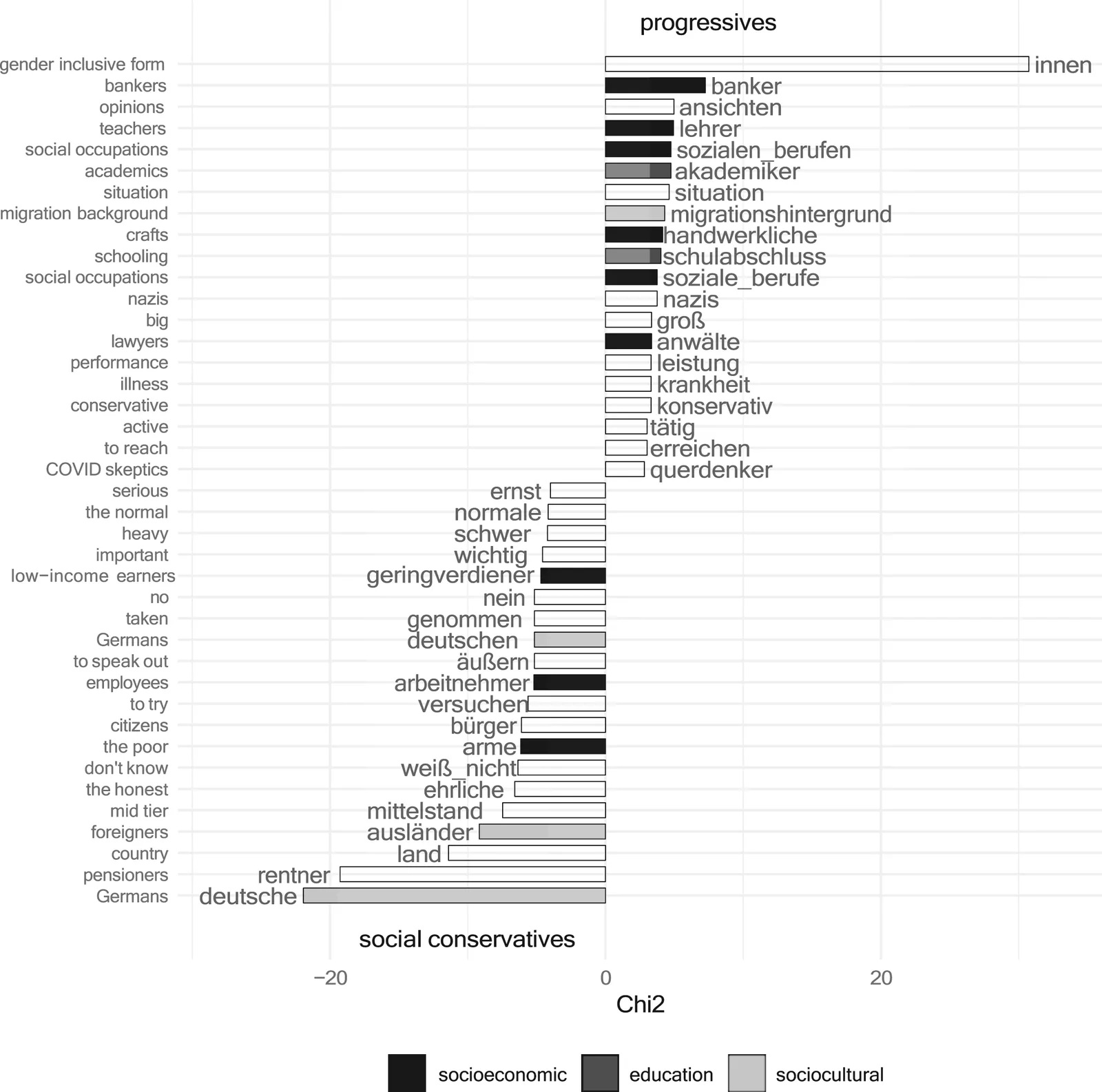
Keyness scores - Subgroup comparison of groups mentioned as losing status. The plot displays the 20 most characteristic words used to describe losing groups, by progressive (top) vs. conservative attitudes (bottom). The more extreme the Chi-squared statistic (= keyness score) on the *x*-axis is, the more characteristic (or comparatively frequent) a term is for one group compared to the other.

In combination with insights from the distinctively perceived status winners, status changes related to migration/race seem to be perceived as zero-sum by social conservatives, with “Germans” seen as losing at the expense of “immigrants”. To assess the existence of zero-sum thinking in a more systematic manner and on the individual instead of aggregate level, we include additional analyses in section C4 in the appendix. Specifically, we regressed mentioning Germans or natives as status losers on mentioning migrants or People of Color as winners, controlling for individual sociodemographic characteristics and attitudes. These analyses show that the same socially conservative respondents who mention Germans as losers also mention migrants as winners, while progressives are much less likely to mention Germans or white people as losers, or migrants as winners. In contrast, for gender, there is no such prominent reactionary zero-sum thinking. Mentions of women as status winners and men as losers are not more prominent for either ideological group and men are overall rarely perceived as losers (Figure C7). The acknowledgment that women are gaining recognition thus appears to be less directly linked to zero-sum narratives in respondents’ minds.

Coming to perceptions of status losers among progressives, “migration background” is the only explicitly sociocultural term that sets apart progressives’ description of social status losers. This mirrors right-wing conservatives’ responses on this topic, albeit reflecting a very different sentiment. Further, whereas mentioning gender, sexuality, and race is distinctive for progressives’ descriptions of status winners, mentioning the opposite sides of these sociocultural developments (men, heterosexuals, no migration background) as status losers does not distinguish progressive from conservative views.

Interestingly, education (“academics”, “schooling”) is only a distinctive category for progressives’ views of status losers. This might seem surprising given the importance of education in knowledge economies. Looking at the context of these mentions reveals that progressives perceive a declining value of tertiary degrees in times of educational expansion. Additionally, they mention “schooling” in the context of groups with no or lower qualifications, thus perceiving downward shifts by people with low education over the past decades.

Finally, progressives mention some political outgroups as status losers (“Nazis”, “conservative”, “COVID skeptics”), indicating that they perceive their own progressive values to be on the rise. Social conservatives seem to agree and mention “Greens” as status winners. Thus, politics and socially accepted norms and values seem to be rather uniformly perceived – at the time of fieldwork in 2022 – as changing in a more progressive direction.

To corroborate the keyness scores on differing status perceptions of social conservatives and progressives, we analyzed individual responses using logistic regression models. We assess whether respondents mention sociocultural and socioeconomic terms and further examined mentions of race and gender/sexuality to explore heterogeneity *within* the sociocultural category.^21^ The results, depicted in Figure 6 show the predicted probabilities of mentioning the different dictionary (sub-)dimensions for progressives and social conservatives, controlling for individual-level factors like education, income, and gender (Panel A). It confirms that progressives are more likely to mention sociocultural groups as status winners compared to social conservatives. This difference in perceptions of status winners is particularly driven by progressives mentioning terms related to gender and sexuality more often than social conservatives. Racial groups are similarly infrequently mentioned as winners by both ideological groups.^22^

**Figure 6.**
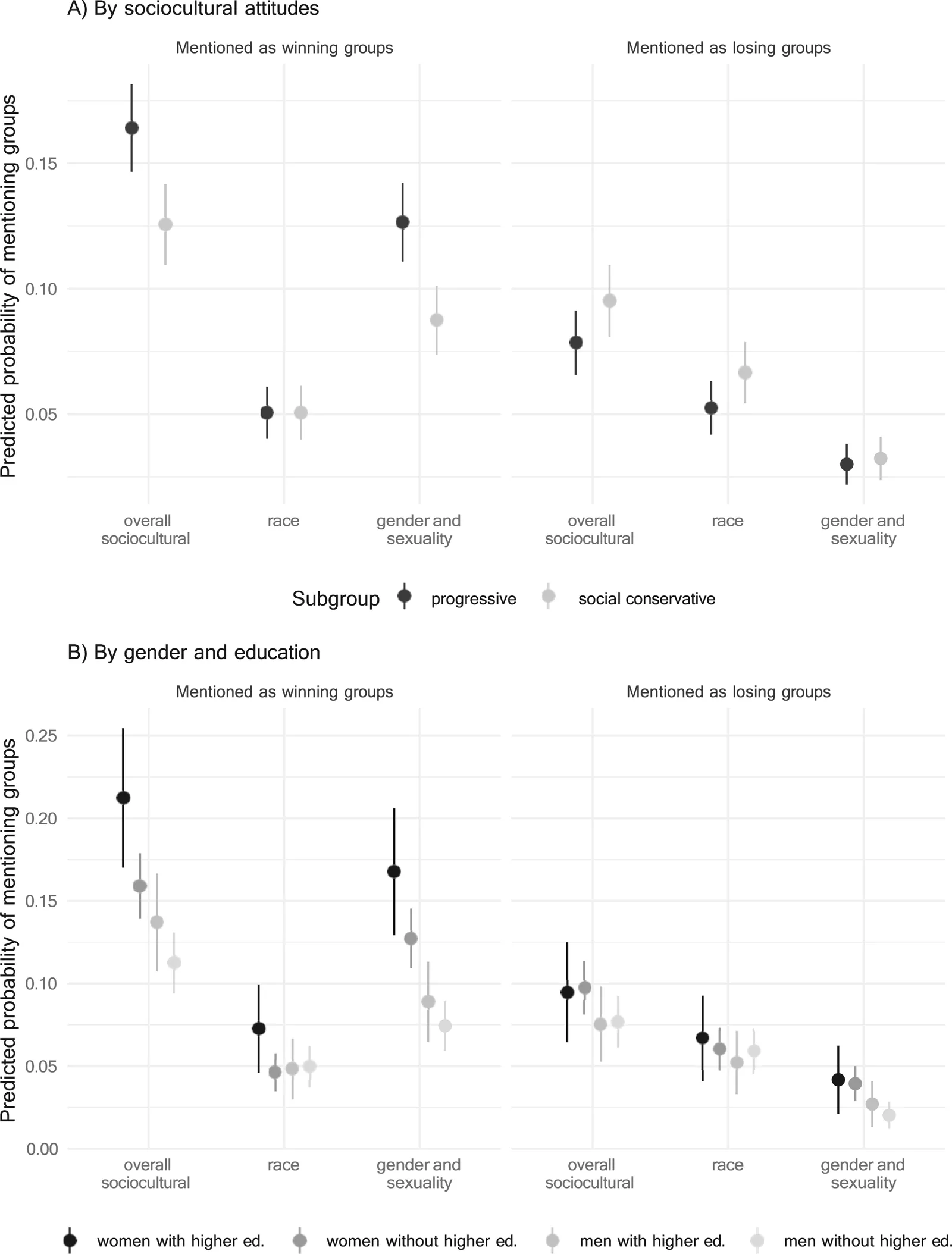
Predicted probabilities of mentioning sociocultural groups as winning or losing, by attitudinal subgroup (top) and education and gender (bottom). Marginal predicted probabilities of a binomial generalized linear model (calculated using Arel-Bundock et al. (2024)), controlling for redistribution attitude, gender, age, education, income, urban/rural residence and migration background. Confidence intervals: 95%, see Appendix tables C3, C4, C5 and C6 for full models.

Regarding perceptions of who is losing status, social conservatives are slightly more likely to mention sociocultural terms overall. This tendency is more pronounced for racial compared to gender and sexuality groups. The differences between attitudinal groups are not significant here. However, using a continuous specification of attitudes, social conservatives are significantly more likely to mention sociocultural and racial groups than progressives (see Tables C3 and C4 in the appendix). We derive from these patterns that subgroup differences regarding perceptions of status losers are smaller and are more related to mentions of race, whereas differences in perceptions of status winners are bigger and more influenced by mentions of gender and sexuality terms.

Next to subgroup differences by ideology, the bottom panel B of Figure 6 also displays how likely women and men with different education levels are to mention sociocultural groups as winners and losers. This shows that sociocultural groups, especially gender and sexuality groups, are predominantly perceived as winners by people who can be considered relative winners themselves. Highly educated women, and also lower-educated women, are significantly more likely to perceive gains of groups like women and LGBTQ+ people, whereas these societal developments do not appear at the top of men’s minds.^23^

To sum up, we find important differences in perceptions of status shifts. Rather than the groups considered to be prone to a cultural backlash (social conservatives and lower-educated men), the people welcoming these changes (progressives and women) are much more likely to saliently perceive and mention status gains by women and LGBTQ+ people. In contrast, social conservatives mention sociocultural groups less often, and if so, mostly when thinking about *losers* of social status. We find that they display zero-sum thinking concerning status shifts due to migration: Social conservatives explicitly connect perceived losses by natives with gains by migrants or People of Color.

It is important to highlight that despite our focus on sociocultural groups for this subgroup comparison, socioeconomic groups are still more prominent in absolute terms for the dynamic questions (Table 1). This holds for both social conservatives and progressives. However, in our regression analysis, we find that progressives are significantly more likely than social conservatives to mention socioeconomic groups when describing both who is gaining and who is losing status (Figure C2 in the appendix).

## Conclusion

This paper provides new evidence on how individuals think about the social hierarchy. Open-ended questions, asking respondents to describe people who are higher and lower in the social order and winning or losing status, provide us with a sense of which groups are at the forefront of people’s mental maps of society.

At a very fundamental level, our results show that respondents are able to articulate a structuralist vision of society, naming specific social groups and not just individual attributes like merit or diligence. Social groups that have been of central interest in the scholarly literature figure prominently in voters’ perceptions. This is the case for women, ethnic, racial, or sexual minorities – but these groups feature in people’s perceptions of the status hierarchy in very specific ways.

Our findings show, first, that socioeconomic reference groups remain central to people’s views of social hierarchies, even in a time when sociocultural group conflicts have become politically more salient. We attribute this to long-established economic standards for social valuation (Lamont, 2019). Especially for perceptions of low/high positions in society, socioeconomic categories are clearly dominant, including references to income, money, wealth versus poverty, as well as elite occupations as opposed to unemployment or low-wage work. Even progressive respondents, who are most likely to perceive and mention improvements in the social position of women and minorities, are overall likely to view social hierarchies largely in socioeconomic terms. This is compatible with evidence that such voters care about *both* sociocultural and socioeconomic inequalities (Abou-Chadi & Hix, 2021; Abou-Chadi, Ha¨usermann et al., 2025).

Second, our results clearly show that we need to consider peoples’ perceptions of dynamic changes to the social hierarchy separately: while socioeconomic categories are important here as well, *this* is where much-discussed sociocultural groups such as women, ethnic minorities, or LGBTQ+ groups come into play. According to our respondents’ perceptions, social hierarchies related to gender, ethnicity, and sexuality have changed, but hardly reversed in absolute terms.

Third, subgroup analyses provide evidence that important aspects of social hierarchies are contested and not equally perceived across different segments of society. Notably, sociocultural groups, such as women and LGBTQ+ people, are distinctively mentioned as gaining status by progressive respondents, whereas social conservatives characteristically mention native Germans as status losers. Importantly, immigrants’ status gains are clearly perceived in a very different light by progressives and social conservatives. Social conservatives’ sense that immigrants win – while natives lose – is the only clear indication we find of changes in social hierarchies being perceived as zero sum. In contrast, progressives do not view gains by people with migration background as happening at the cost of the majority white population.

Generally, while we find evidence of a fully articulated backlash on matters of race and ethnicity among social conservatives, the same cannot be said of gender and sexuality: while women’s social position is widely perceived to be improving, it is not the case that as many people see men as declining, even when we contrast social conservatives and progressives (similarly for sexual orientation) (also see Alexander et al., 2024). Some aspects of a backlash may of course remain hidden or silent in our study, with conservatives resenting but not explicitly acknowledging, say, women’s status gains. That said, our results certainly highlight a point that is sometimes neglected in talk about political backlash to progressive change: how these shifts appear at least as salient and important for progressive voters as they are to voters who might feel threatened by them.

In more practical terms, our findings have clear implications for the empirical study of sociocultural group conflict. Recent research on status anxiety, including highly influential studies, has worked with an item that asks people to place themselves on a social ladder representing the top versus bottom of their society today (Engler & Weisstanner, 2021; Gidron & Hall, 2017, 2020; Kurer, 2020; Oesch & Vigna, 2022). Our analyses based on open-ended survey responses show this to be problematic, at least in terms of interpretations focused on resentment against sociocultural change. We demonstrate that such a question about a static (current) social hierarchy is unlikely to tap into people’s perceptions of how socioculturally defined group positions are changing (see also Anderson, 2025). For instance, women, while widely perceived as gaining status in our sample (as e.g. Gidron & Hall, 2017 suggest), feature nowhere in the most frequent terms with which our respondents describe the top or bottom of society. An approach building on Gest et al.’s (2018) questions about dynamic status change seems more promising, also complementing qualitative work (Damhuis, 2020; Hochschild, 2016; Westheuser, 2021). Our analyses further highlight potential trade-offs between capturing very specific group threats and priming people to think about status shifts that are perhaps not most salient to them in the bigger scheme of things.

Overall, this study based on open-ended survey questions validates a productive research agenda seeking to tie voters’ status concerns to often socioculturally mobilized electoral conflicts, but it refines the extent to which, where, and how sociocultural aspects of (changing) social hierarchies can be expected to be perceived by voters. We expect our results from Germany to be indicative of voter perceptions in other West European countries, since Germany has experienced all the major structural and economic changes (e.g. educational expansion, the shift towards a knowledge-based economy, a rise in economic inequality) and waves of political mobilization (the early success of the Greens, the rise of a far right, mobilization around gay and women’s rights, etc.) that have recently affected and shaped the social fabric of advanced democracies. If anything, concerns about changing ethnic or racial hierarchies might be articulated even more clearly in other European countries, since social desirability might prevent some Germans from clearly talking about ethnicity due to Germany’s Nazi past.

A larger caveat perhaps concerns the timing of our survey, fielded in 2022. While social transformations were already highly politicized, at least the long-term direction of change and achievements already won in the realms of gender equality or minority protection perhaps seemed safer than they do now, from a progressive perspective. Notably with the second presidency of Donald Trump in the US, far right efforts to translate a cultural backlash into policy output and outcomes have become more emboldened and tangible. With more social and political actors openly contesting or tacitly dropping progressive stances, the question is whether and when this development would start to show up in voters’ descriptions of who is gaining/losing respect and recognition in society.

## Supplemental Material

Supplemental Material - Narratives of Backlash? Perceptions of Changing Status Hierarchies in Open-Ended Survey Responses Supplemental Material for Narratives of Backlash? Perceptions of Changing Status Hierarchies in Open-Ended Survey Responses by Magdalena Breyer, Tabea Palmtag, and Delia Zollinger in Comparative Political Studies

## Consent for Publication

All survey respondents were informed about the nature of the study, its authors, and its duration and had to provide informed consent before starting the online questionnaire.

## Data Availability

Replication materials and code can be found at: Breyer et al., 2025. “Replication Data for: Narratives of Backlash? Perceptions of Changing Status Hierarchies in Open-Ended Survey Responses.” Harvard Dataverse. https://doi.org/10.7910/DVN/XUNKG6.

## Notes

1. For just a few examples from the US, the UK, or Germany see Chokshi (2018) and Edsall (2020) in the New York Times, Nachtwey (2016) in Die Zeit, or Gest (2017) in The Guardian.

2. For instance, as indicated in Figure 1, women have benefited disproportionately from expanding higher education and the growth of high-skilled professions, but simultaneously remain overrepresented in precarious, low-paid, atypical employment. And while certain lower-status and lower-skilled jobs may well be associated with, e.g. workers of migrant background – not just in reality but also in people’s minds - immigration has arguably been made salient primarily through a lens of *changing* status and opportunities for mobility among ethnic minorities.

3. We theorize and later empirically categorize all references to occupations as primarily“socioeconomic”. (Occupational) class also has cultural elements, and it is well-established that horizontal class divisions help categorize the core electorates of the far right and new left parties opposed over sociocultural politics (Oesch, 2006; Oesch & Rennwald, 2018). Nevertheless, in the context of our questions about a vertical hierarchy, we opt to treat references to managers and entrepreneurs as analytically close to the traditional class conflict. Below, we discuss more ambiguous cases such as mentions of “workers” or “policemen” (which has a law and order connotation).

4. These characteristics of course further intersect in ways highly relevant to status.

5. We have already touched upon the relatively changing importance of a university degree, the shifting social position of an old – majority-ethnic, white – working class, or growth in income inequality; some of which is reflected in Figure 1.

6. I.e., voters who adopt more conservative positions on “second dimensions” issues such as gender equality, migration, or LGBTQ+ rights; who have also been labeled “traditional-authoritarian-nationalist (TAN)” or “particularist”; have a higher likelihood of supporting the far right; and who tend to be more lower-educated, white, and male (e.g. Bornschier, Haffert, et al., 2024; Hooghe & Marks, 2025; Kriesi et al., 2008).

7. In the conclusion, we will discuss how the social changes summarized in Figure 1 have come under ever more open attack since we first formulated these expectations and fielded our survey in 2022. Progressive social change has of course long been highly contested, but with the second Trump presidency, far right efforts to blatantly dismantle and reverse progressive social transformations have arguably reached another level. These shifts, again, could become reflected in voters’ perceptions of changing status.

8. More information on the survey flow, attention check and all modules included in the survey can be found in Appendix A. The study was approved by the responsible Ethics Board.

9. Replication materials and code can be found at Breyer et al. (2025).

10. Respondents were first asked to give information on their own status and status trajectory before answering open-ended questions about their perceptions of (changing) social hierarchies. Two items provided additional context for the open-ended item: the first asked respondents to place themselves on a social ladder ranging from the “bottom” (1) to the “top” (10) of society for the present. The second asked, “Now please imagine that the scale represents the situation in Germany 30 years ago. Where do you think someone like you would have stood 30 years ago?” The difference between present and past positions was then used in the open-ended question wording and reminds respondents of their perceived trajectory for people like them. We did this to clarify the concept of the ‘changing position in the social hierarchy’ for respondents. Relating our open questions to the often used closed-ended “social ladder” question also allows us to cumulatively build on and speak to existing research, including on matters of measurement.

11. We included a third open-ended question on status deservingness to capture respondents’ normative views and attitudes toward status hierarchies which are not part of this analysis.

12. These items read: “Overall, immigration is bad for German society.”; “European integration has gone too far.”; “The bottom line is that the family suffers when the wife works full time”.

13. For all open questions, respondents were required to write at least one character to proceed in the questionnaire.

14. Appendix B includes details on preprocessing.

15. We focus on the socioeconomic, sociocultural and educational dimensions in our results, but Table C1 in the Appendix also shows results for further dimensions focusing on politics and attributes/personality traits.

16. In Appendix A4, we include some more exemplary quotes. For the bottom of society, respondents for example mention poverty and difficult life prospects.

17. We categorized all professions as occupations and thus as socioeconomic in our dictionary. Some occupations like policemen carry more social or cultural connotations (like law and order) than others. However, further subcategorizations for the occupations would have become arbitrary, since all occupations carry some social prestige evaluations (Chan & Goldthorpe, 2004). Thus, we rather capture a lower bound of the sociocultural salience here, focusing on core elements of “backlash” narratives.

18. Table C2 in the appendix breaks down these results by dictionary subdimensions.

19. This does not mean that social conservatives do not mention e.g. women as gaining status at all, but that progressives do so relatively more frequently.

20. In Germany, race as a category is seldom explicitly politicized, unlike migration. The racial and ethnic connotations of “migrant” stem from the historical recruitment of “Gastarbeiter” and restrictive jus sanguinis citizenship laws, linking migration to ethnic identity. In contrast, the UK and US, with longer histories of diverse immigration and jus soli citizenship, focus more on specific racial and ethnic identities rather than a broad categorization of migrants. This is reinforced by public discourse and integration models that emphasize multiculturalism in the UK and US, unlike the German focus on cultural adaptation. Consequently, mentions of migration background in Germany often serve as coded references to race and ethnicity (Elrick and Farah Schwartzman 2015), as shown by subgroup analyses contrasting these groups with “German” natives.

21. We assess mentions of dictionary dimensions as binary outcomes, meaning that a response is coded as 1 if one or more terms of a (sub)dimension appear and zero if not.

22. To substantiate these analyses based on categorizing respondents as either ‘progressive’ or ‘socially conservative’, we also ran the same logistic models with continuous and squared GAL-TAN attitude variables instead of the categorical variable. These models are presented in Figure C3 and in Tables C3, C4 and C5 in the appendix. They show very similar results. The specification with the polynomial points to a slight U-shape of the association for perceptions of gains, but the confidence interval for the social conservative end of the scale is very large.

23. We included further subgroup comparisons by sociodemographic characteristics in the appendix, namely by respondents’ migration background and by their gender and age. We do not find differences in status shift perceptions by migration background (Figure C4), this could partly be due to the imperfect nature of this indicator for ethnic background. The patterns by gender and age (Figure C5) show that young men are more likely than older men to mention sociocultural groups as winning. This tendency is in line with other work showing that younger men are more prone to reactionary gender positions (Kim & Kweon, 2022; Off et al., 2022), as we see that this topic has a certain salience for them. However, young men are still significantly less likely to mention gender groups than young women. An additional keyness analyses shows that young men do perceive gains of “feminists” on rank 7 in terms of keyness, but the salience of gendered groups is much higher for young women, who mention “women” and “mothers” on ranks 1 and 3 (Figure C6).
