# Supplementary material for: Narratives of Backlash? Perceptions of Changing Status Hierarchies in Open-Ended Survey Responses
Source: Comp Polit Stud. 2025 Nov 11;59(13):2928–63. doi: 10.1177/00104140251392533 (PMC13630304; doi:10.1177/00104140251392533)
Supplement: Supplementary file 1 [file sj-pdf-1-cps-10.1177_00104140251392533.pdf]

Online Appendix

Narratives of Backlash? Perceptions of Changing Status Hierarchies in Open-Ended Survey Responses

Magdalena Breyer, Tabea Palmtag, Delia Zollinger

September 16, 2025

All replication files (data and code) are available online in the Harvard Dataverse under the DOI: 10.7910/DVN/XUNKG6

Contents

A. Survey **2**

A1. Sample characteristics . . . 3

A2. Open question wording . . . 4

A3. Consistency between two surveys . . . 6

A4. Exemplary Quotes . . . 11

B. Text analysis of open questions **13**

B1. Deleted terms . . . 14

B2. Compound Terms . . . 15

B3. Dictionary . . . 20

C. Additional results and regression tables **31**

C1. Results for medium status group (static) . . . 31

C2. Salience of dictionary (sub)dimensions . . . 32

C3. Individual-level analysis of dictionary mentions . . . 34

C4. Tests of zero-sum thinking . . . 44

A. Survey

We fielded our survey in two (non-panel) waves, with 1'941 respondents interviewed in January/February 2022 and another 1'871 in September 2022 (a slight change in wording between the two surveys is discussed in section A2).

The survey company Respondi recruited our respondents from their online access panel and reimbursed them based on the length of the survey. All respondents were informed about nature and goals of the study and only those providing voluntary and informed consent proceeded in the questionnaire. A pre-test (n of about 300, included in the total sample size) was conducted before the fielding of the survey and confirmed that respondents understood the open questions correctly. For the attention check, we instructed respondents to select a specific value on a seven-point-scale within a battery of items on political preferences (randomized order). Respondents who failed to select the correct value were screened out directly.

The survey started by asking about a first set of sociodemographics (including education and gender for population-representative quotas), followed by social identities, political attitudes, and subjective social status, after which we asked the open questions (see next section), vote choice and finally another set of sociodemographics (including occupation, income, and migration background).

A1. Sample characteristics

Table A1: Sample characteristics

		N	Percent
Gender	woman	1934	50.81
	man	1863	48.95
	nonbinary	9	0.24
Education	low education	776	20.39
	medium education	2028	53.28
	high education	1002	26.33
Income	low income	1501	39.44
	medium income	1115	29.30
	high income	874	22.96
Age	18-39 years	1400	36.78
	40-59 years	1451	38.12
	above 60 years	894	23.49
Migration background	no	3259	85.63
	yes	542	14.24
Residence	countryside	888	23.33
	suburb/small city	1897	49.84
	big city	1017	26.72
Vote choice	Social democrats (SPD)	863	22.67
	Christian democrats (CDU/CSU)	546	14.35
	Green party	601	15.79
	Liberal party (FDP)	427	11.22
	Alternative for Germany (AfD)	347	9.12
	Left party (LINKE)	246	6.46
	other party	308	8.09
	All	3806	100.00

Vote choice: party voted in 2021 (or would have voted, if non-citizen); Residence: selfreported degree of urbanity/rurality; Migration background: respondent or at least one parent born abroad; Income: equivalized according to household size.

A2. Open question wording

This section lists the English translations of the original German language open questions.

1. Introduction page:

Three open questions follow, where you can freely formulate your answers. It is particularly interesting for our research how you think about the social position of different groups in your ***own words****. When we determine our own social position, as you did in the previous questions, we usually already have a picture of society in mind. That's what the next questions are about.*

2. Dynamic question:

How much groups are recognized and respected in our society may change over time. You yourself indicated in the previous questions that the social position of people like you has improved/not changed/worsened (depending on subjective social status answers) *over the last 30 years.*

Now think about the people who have tended to gain recognition compared to the past. *How would you describe these people? What kind of characteristics, lifestyles, and opinions do these people have?*

Now think about the people who tend to get less respect compared to before. *How would you describe these people? What kind of characteristics, lifestyles, and opinions do these people have? Just write down what comes to your mind, this is not about right or wrong. Please describe them in a few key words or short sentences.*

Groups winning recognition: _

Groups losing recognition: _

3. Static question:

Let's get back to our society today. Not all social groups receive the same amount of respect and recognition. You have placed yourself in more of a top/medium/bottom (depending on subjective social status answer) *social position.*

How would you describe groups that are currently more on the ***upper edge*** *of society, who receive a lot of recognition? How would you describe these people? What characteristics, lifestyles, and opinions do these people have? Please describe them in a few key words or short sentences.*

-

*And how would you describe groups that are in the **middle** of society and tend to get an average amount of respect?*

-

*And how about groups that tend to be at the **lower edge**, who tend to get little recognition?*

-

Note: This is the final question wording from our follow-up survey. In the first wave, for the static question, we asked for the top/middle/bottom positions without specifying the terms "recognition and respect". In the second survey, we introduced these questions about high/low/middling status groups with "Now let's come back to the present situation. Not all social groups equally receive social recognition and respect". This addition in the follow-up survey allowed us to make sure that the mention of "recognition and respect" does not fundamentally change the responses that people give across our dynamic and static open-ended status questions. The results in Appendix A3 indicate that the answers across these slightly different question wordings are extremely similar.

A3. Consistency between two surveys

Our first (N of 1'941) and second survey wave (N of 1'871) varied slightly in the question wording for the static open questions (see Appendix A2). The results in terms of salient status groups are very similar, as shown in the absolute frequency plots below. For each question, results are distinguished by survey wave. The consistency of results for the dynamic perceptions question are shown as a reference point.

Figure A1: High status groups - absolute frequencies

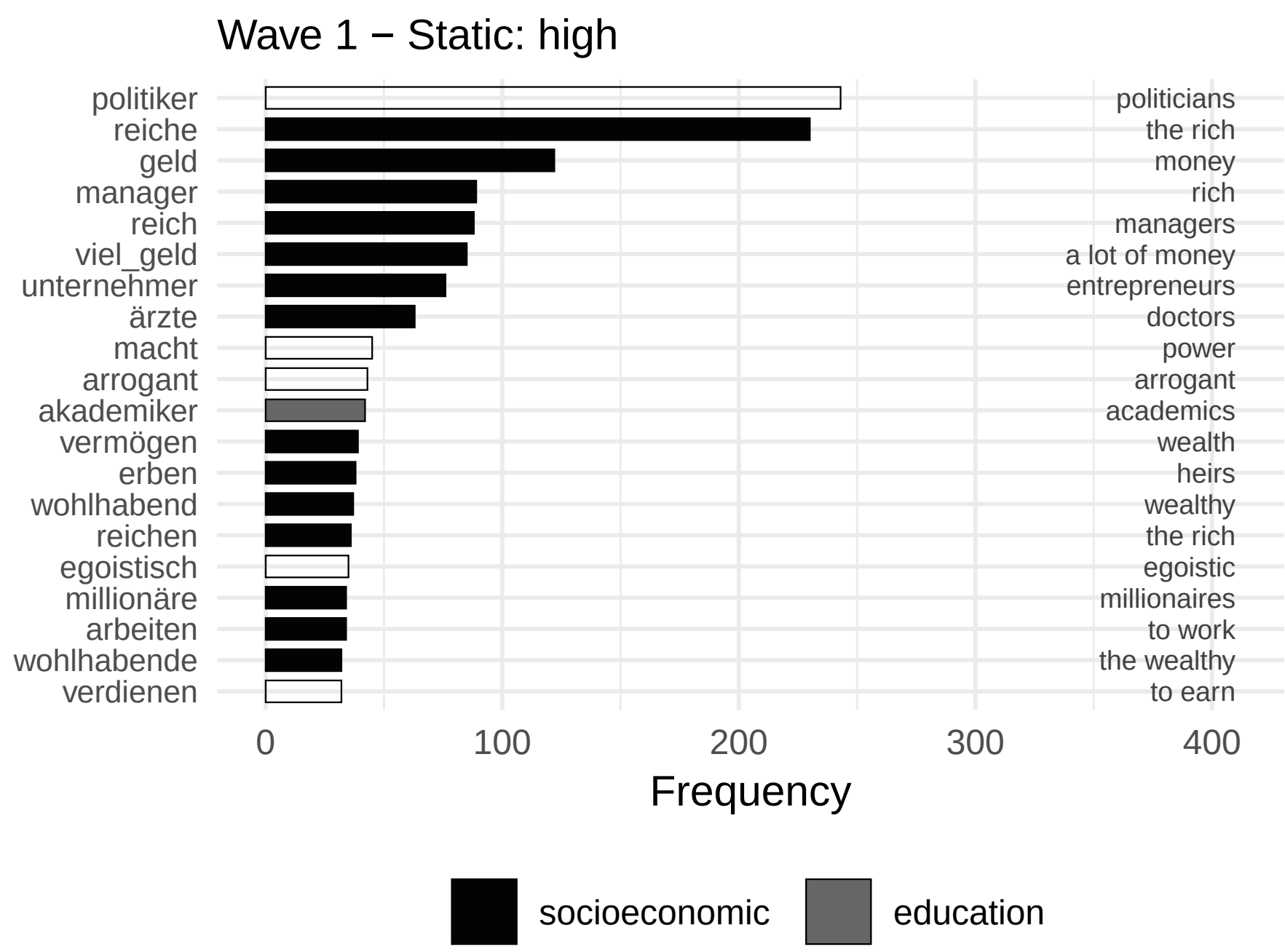

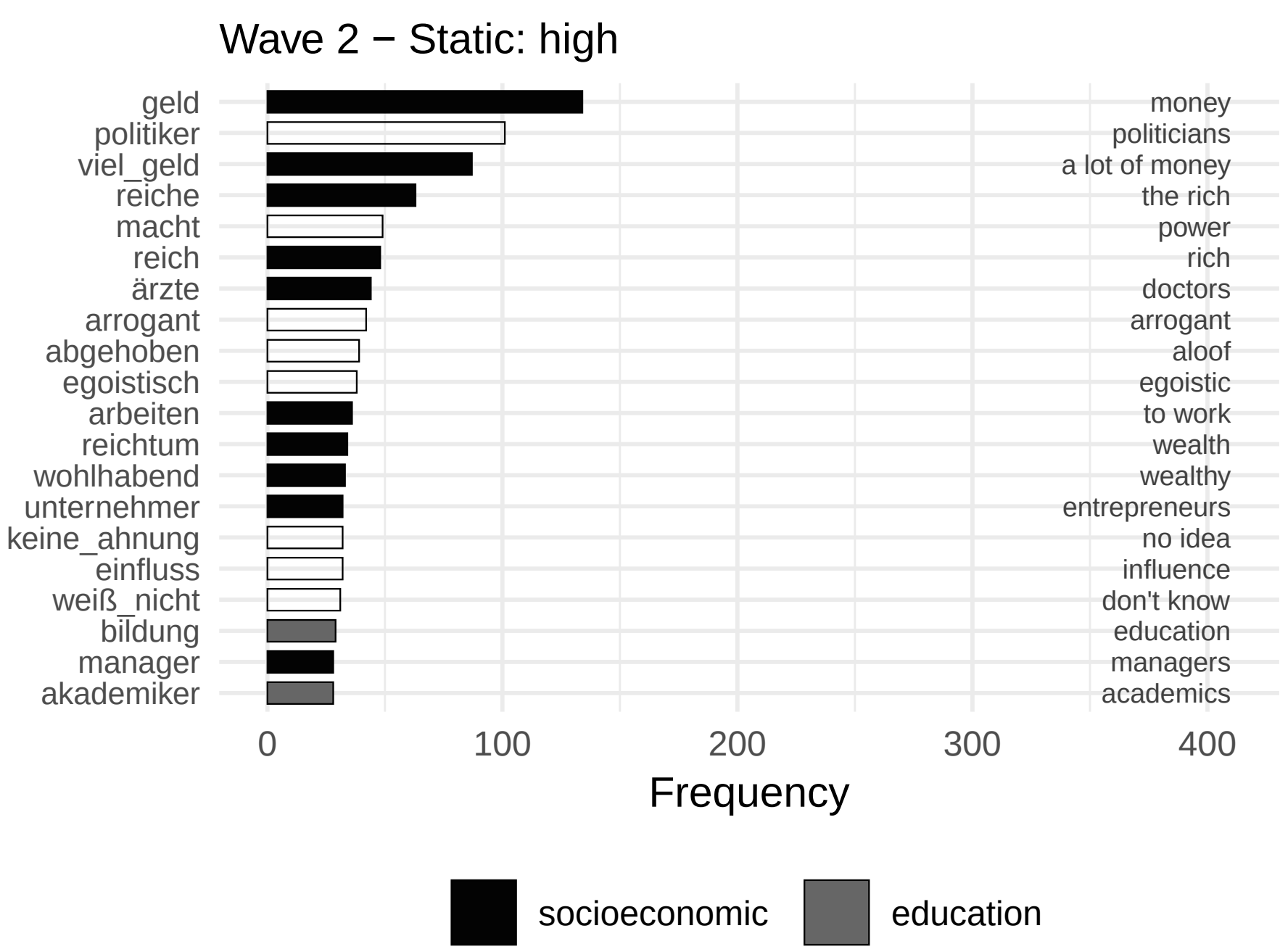

Figure A2: Low status groups - absolute frequencies

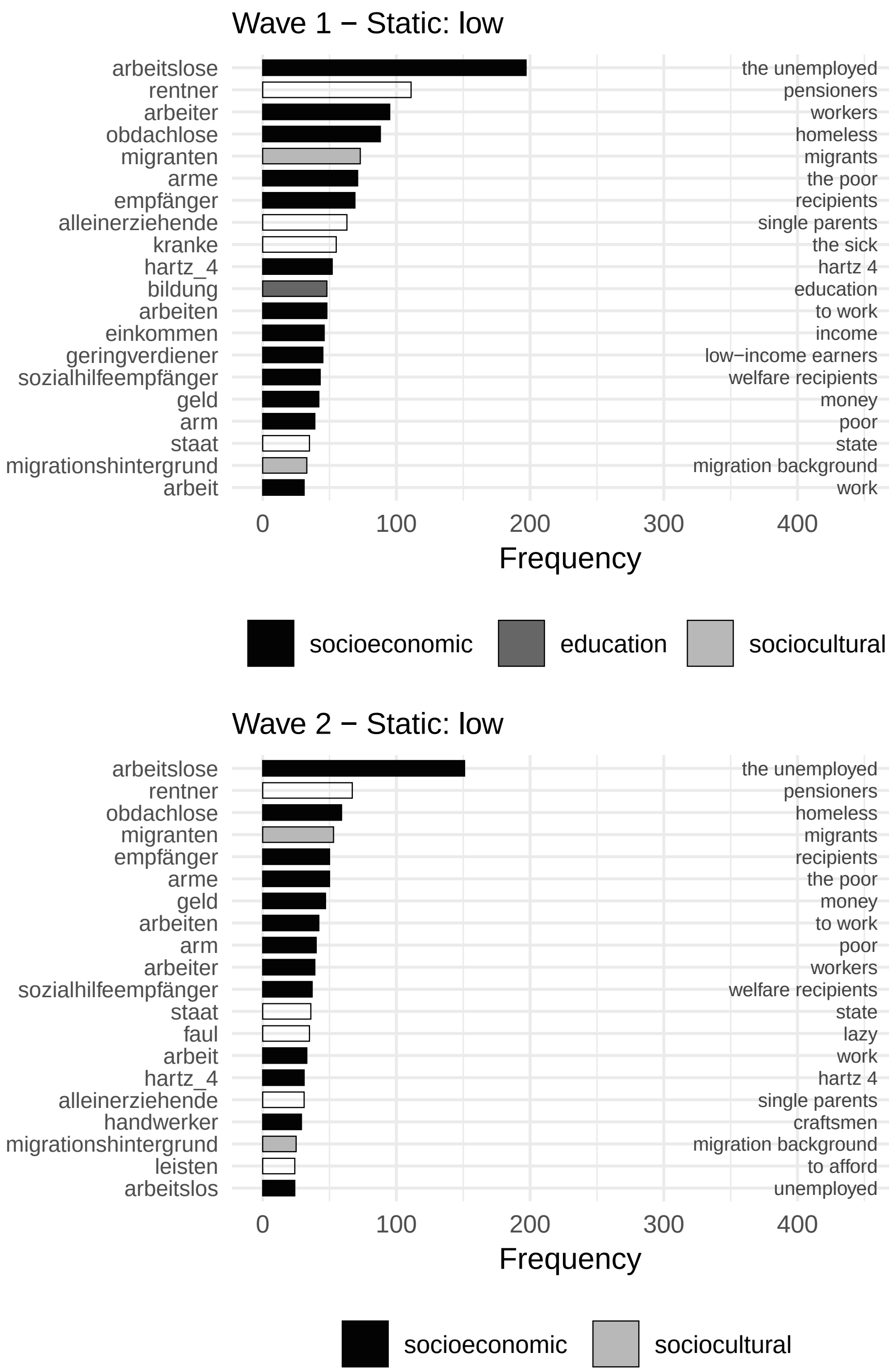

Figure A3: Winning status groups - absolute frequencies

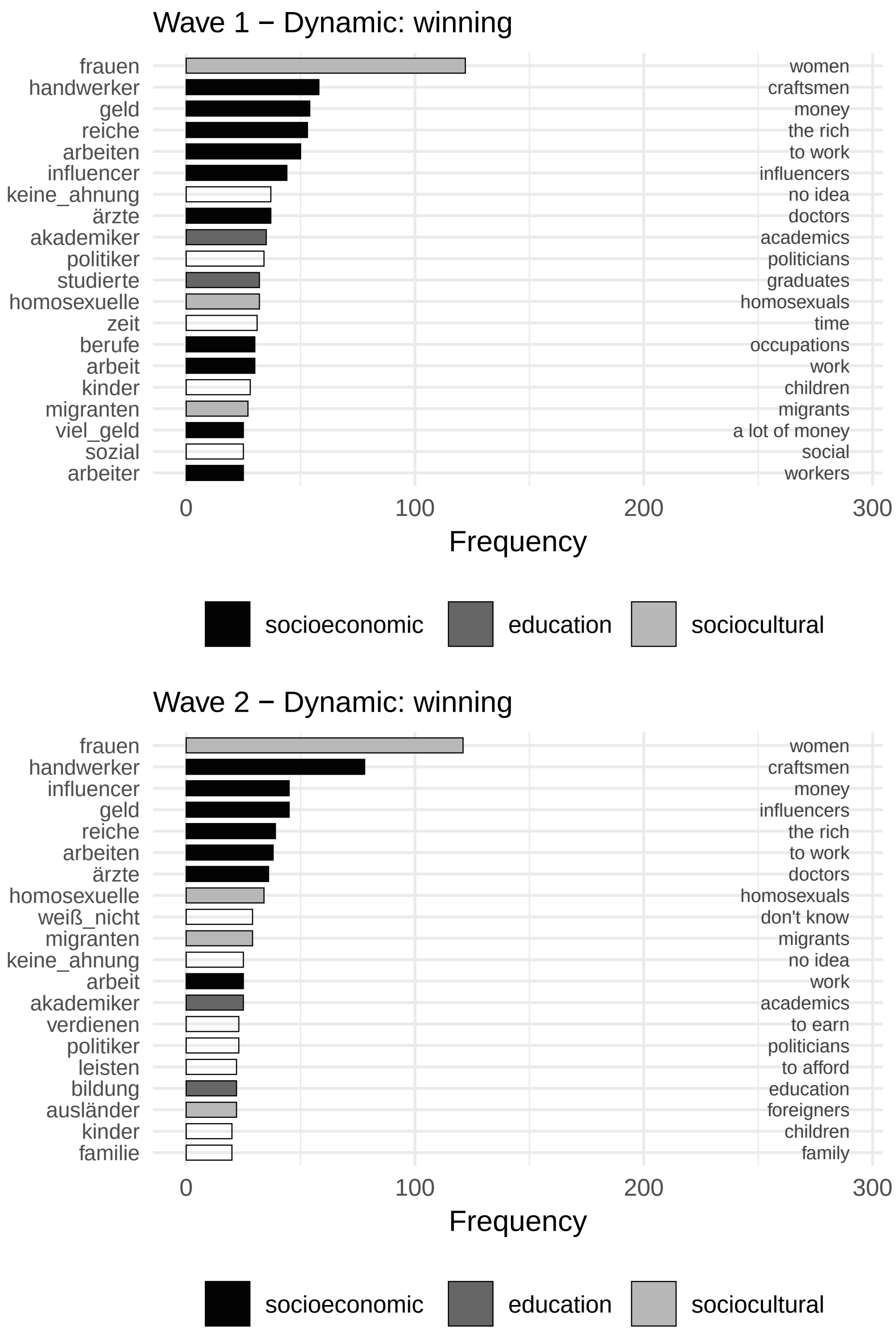

Figure A4: Losing status groups - absolute frequencies

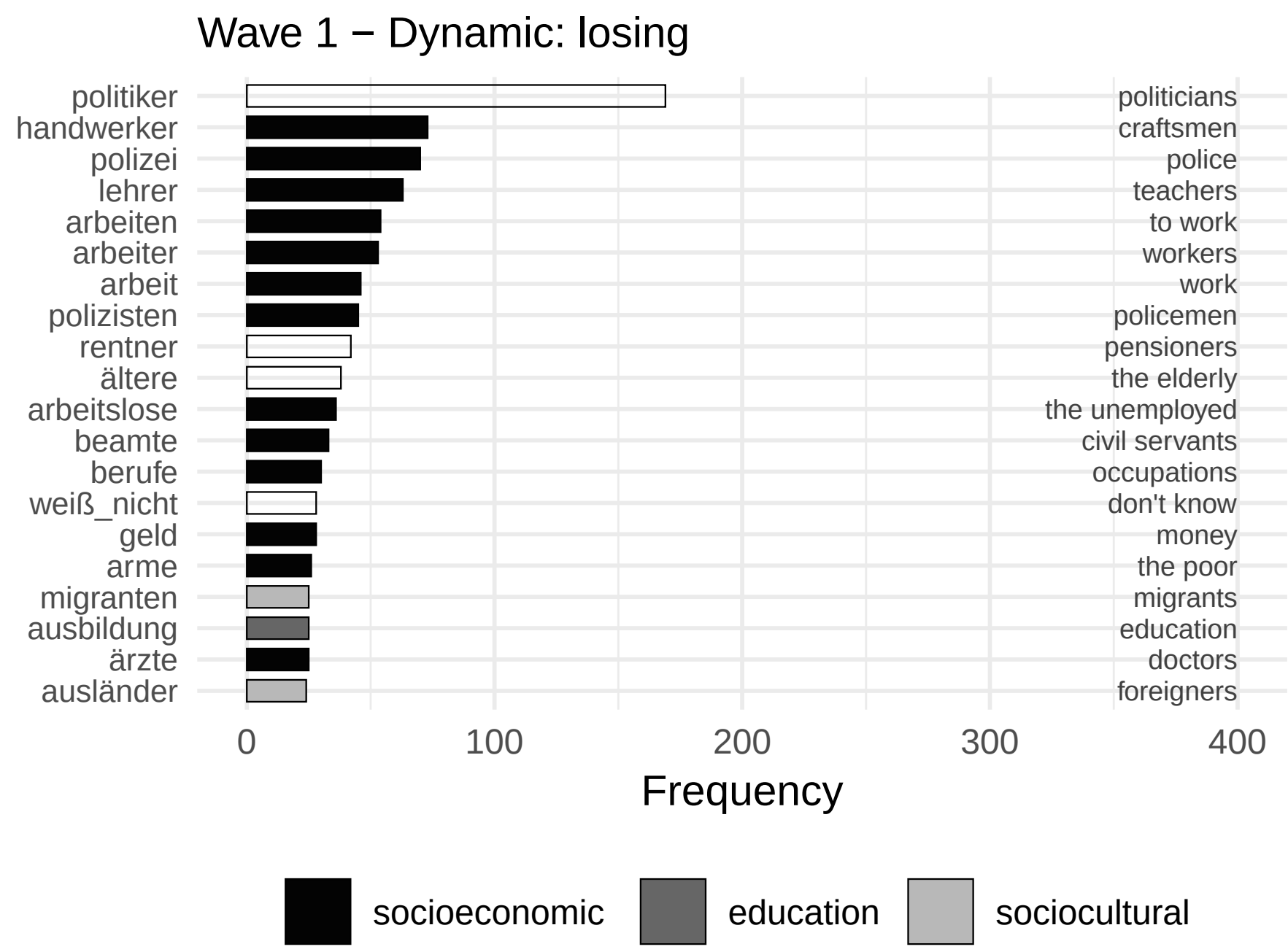

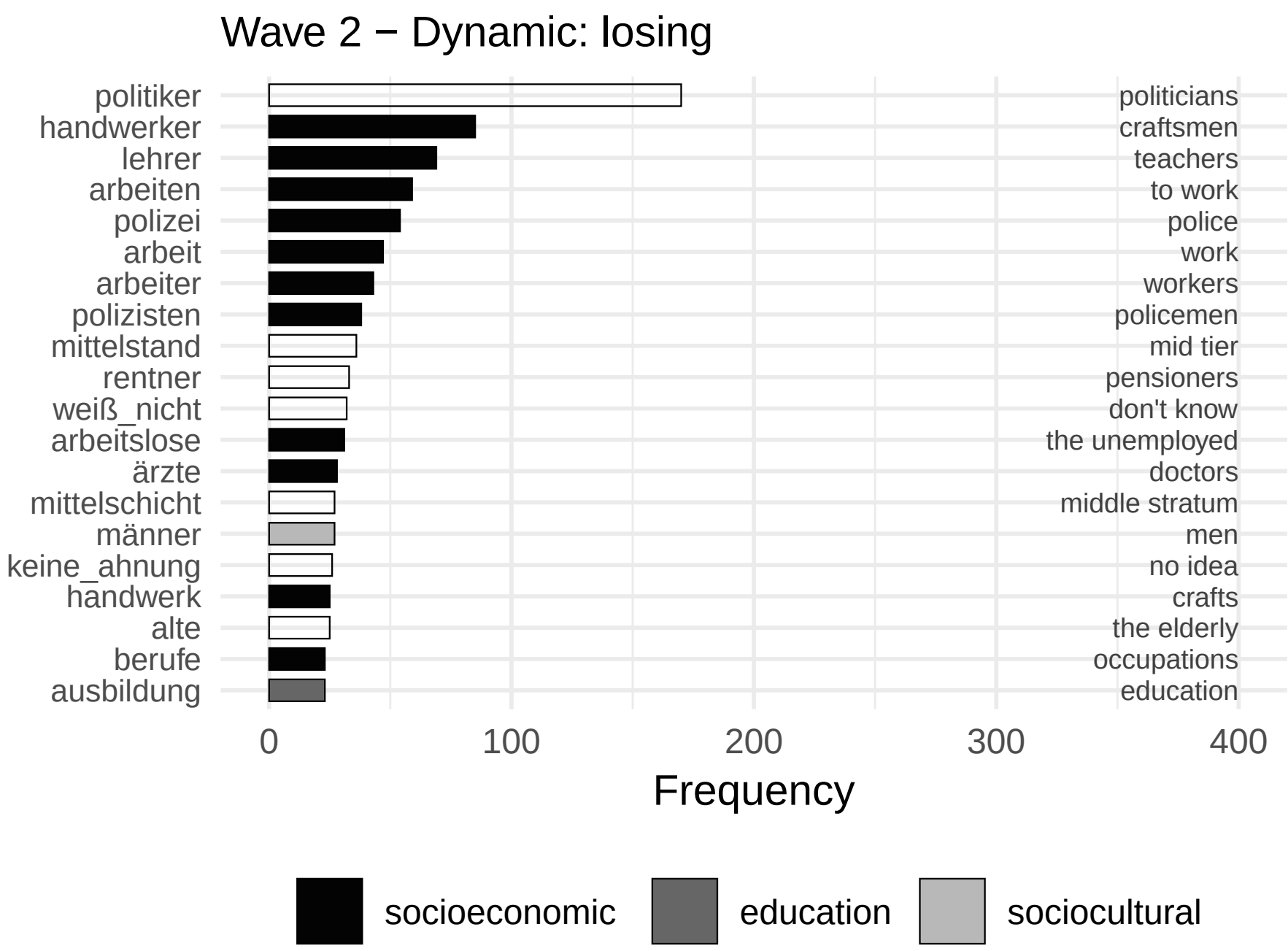

A4. Exemplary Quotes

Table A2: Examples of open answers - static questions

Respondent	High in recognition	Low in recognition
62-year old woman, low education	"They are fortunate and can afford everything."	"Poverty, not accepted in society. No prospects. Living from day to day. No motivation."
29-year old woman, high education	"Superficial life, lots of consumption and possessions"	"Poor, lack of prospects, a difficult situation to work your way up"
68-year old man, medium education	"These are the truly wealthy, millionaires, entrepreneurs, and owners of large companies. They live in their own world."	"Many workers, unskilled laborers, migrants, unemployed people, and some retirees. These groups have below-average incomes and are partly dependent on social benefits."

Table A3: Examples of open answers - dynamic questions

Respondent	Winning recognition	Losing recognition
62-year old man, low education	"In my experience, it's people who have more money. I find that superficial, but unfortunately money still plays the most important role today."	"Conversely, these are people who don't have enough money. And unfortunately, I think they are becoming increasingly stigmatized."
41-year old man, medium education	"Gay people, lesbians, green communists, incompetent politicians, migrants, ignorant people"	"The skilled and hard-working craftsmen"
35-year old woman, high education	"Women, including women who work and have families, members of the LGBTQ community"	"White men who have obtained positions not always through their abilities, but through their white, heterosexual masculinity."
52-year old woman, medium education	"Craftsmen are in demand like never before"	"Teachers, police officers, politicians. The population no longer wants to be told what to do. Respect is waning."
55-year old man, medium education	"Qualified immigrants who help us move forward and achieve something. (Founders of Biontech, or doctors and nurses)"	"There are differences between migrants who do not work and cannot or do not want to contribute to society. Don't get me wrong, we urgently need immigration, but it must be qualified and targeted!"

B. Text analysis of open questions

Our pre-processing of the text data included the following steps: First, we removed six respondents who answered the open questions in English language. Per question, we then removed answers with fewer than three characters to exclude non-answers. Second, we removed punctuation and symbols and transformed all tokens to lower case. Third, we analyzed each question separately for frequent collocations (combinations of 2 or 3 words) and included meaningful collocations as compounded tokens. For example, “social occupation” was included as a compound. Last, we removed stopwords and other terms that carry little content, especially words appearing in the question wordings themselves, such as “recognition”, “people”, “today” (see Table B1 for deleted and Appendix B2 for compounded terms). Some terms only appear in the static question wording (surveyed after the dynamic ones). Thus, we exclude these terms only for the answers to the static questions (see Table B2).

B1. Deleted terms

Table B1: Terms deleted in pre-processing of the entire text corpus

‘	gestellt	laufen	u
30	gewinnen	leben	und_co
ahnung	gewonnen	lebensweisen	u_ä
allgemein	geworden	legen	u_u
anerkannt	gibt	leute	überhaupt
anerkennung	gilt	mal	verbessert
befinden	große	man	verfügen
beispielsweise	großer	manchmal	verlieren
bekommen	großen	mehr	verliert
bereiche	gruppe	meinung	verloren
besser	gruppen	meinungen	viel
bzw	gut	meist	vielen
dabei	gute	menschen	weiß
dafür	gutes	mich	weiss
darunter	habe	müssen	wenig
davon	halten	niemals	weniger
denen	heute	obwohl	will
denke	heutigen	oftmals	wohl
denken	heutzutage	personen	wollen
desto	hohe	position	wurde
doch	hohen	positionen	wurden
dh	hoher	respekt	z.b
d.h	höher	respektiert	zb
eher	ihre	schon	z_b
eigentlich	ihrem	sehe	
eigenschaften	ihren	sehen	
eindeutig	ihrer	sei	
einmal	ja	sicherlich	
erfahren	je	siehe_oben	
erhalten	jedoch	sowie	
etc	jemand	stark	
evtl	jahre	stehen	
finde	jahren	steht	
früher	jahrzehnte	stellen	
frage	jetzt	teil	
ganzen	k.a	teils	
gar	kommen	teilweise	
geht	kommt	tendenziell	
gerade	können	unserer	
gesellschaft	lassen	usw	

Table B2: Additional terms deleted in pre-processing for the static questions

rand	oben	oberen
mitte	mittlere	unteren

B2. Compound Terms

Table B3: Compounds Winning

viel geld
sozial* berufe
sozial* medien
social media*
soziale* bereiche*
soziale* netzwerke*
soziale* herkunft
gut* *bildung
besser* bildung
gute* einkommen
gute* hause
weiß nicht
weiß ich nicht
weiß es nicht
kein* ahnung
kann ich nicht sagen
nicht die geringste ahnung
kene ahnung
weis* nicht
fällt dazu nichts
fällt mir
fällt nicht*
fällt nix
keine angabe
keine angaben
z b
u u
u ä

Table B4: Compounds Losing

einfache* arbeiter
einfache* leute
wenig geld
soziale* berufe*
sozial schwache*
hart arbeitende*
har*z 4
hartz iv
handwerklich* berufe*
normale* arbeiter
viel geld
nicht viel geld
mal so viel geld
oben herab
öffentliche* dienst
weiß nicht
kein* ahnung
kann ich nicht sagen
nicht die geringste ahnung
kene ahnung
weis* nicht
fällt dazu nichts
fällt mir
fällt nicht*
fällt nix
siehe oben
keine angabe
keine angaben
z b
u u
u ä

Table B5: Compounds High

viel geld
mehr geld
sich * leisten
hohe* einkommen
reich geboren
keine sorgen
gut* einkommen
was besseres
gut* *bildung
finanziell *gesichert
finanziell unabhängig
über leichen
vitamin b
höhere* dienst*
öffentliche* dienst
eigene* vorteil
auf kosten
weiß nicht
kein* ahnung
kann ich nicht sagen
nicht die geringste ahnung
kene ahnung
weis* nicht
fällt dazu nichts
fällt mir
fällt nicht*
fällt nix
keine angabe
keine angaben
z b
u u
u ä

Table B6: Compounds Medium

wie ich
gut* *bildung
leisten können
nach oben
gerade so
mittler* einkommen
gut* einkommen
runden kommen
sozial engagiert
hart arbeitend*
genug geld
mit dem strom
eigene meinung
9 to 5
klein unternehmer
öffentliche* dienst
mittlerer dienst
höhere* dienst
weiß nicht
kein* ahnung
kann ich nicht sagen
nicht die geringste ahnung
kene ahnung
weis* nicht
fällt dazu nichts
fällt mir
fällt nicht*
fällt nix
keine angabe
keine angaben
z b
u u
u ä

Table B7: Compounds Low

I	II
wenig geld	keine *bildung
einfache* arbeiter	mangelnde bildung
wenig geld	geringe* bildung
soziale* berufe	nicht arbeiten
sozial schwache*	in den mund
weiße männer	auf der straße
hart arbeitende*	ohne ausbildung
ältere* menschen	nicht arbeiten
har*z 4	keine arbeit
hartz iv	ohne arbeit
handwerklich* berufe	auf kosten
viel geld	weiß nicht
nicht viel geld	kein* ahnung
mal so viel geld	kann ich nicht sagen
ohne ausbildung	nicht die geringste ahnung
gering* einkommen	kene ahnung
keine arbeit	weis* nicht
runden kommen	fällt dazu nichts
nicht bereit	fällt mir
alg i	fällt nicht*
alg ii	fällt nix
arbeitslosengeld ii	keine angabe
arbeitslosengeld i und ii	keine angaben
gering verdiener	z b
viele kinder	u u
schlechte bildung	u ä
schlecht bezahlt*	

B3. Dictionary

We classify the terms in our corpus into 6 different dimension for the dictionary analysis: socioeconomic terms, sociocultural terms, terms related to education, politics, attribute words or don‘t know terms. The following tables show these six dimensions and the corresponding terms in the original language of the survey and their English translation.

When categorizing terms that do not obviously fall into one dimension, we checked the context of mentions of the term to help inform our decision. For example, we categorize “social media” and “internet” as socioeconomic, because it was overwhelmingly used to describe occupations like influencers or IT specialists. Where no such context was obvious, we did not categorize the term.

Table B8: Socioeconomic Terms

occupation	occupation (I)	occupation (II)	wealth	wealth (I)
ärzte	krankenschwestern	erwerbstätige	reiche	sozialschmarotzer
manager	erzieher	arbeitsmarkt	reich	geringem_einkommen
unternehmer	altenpfleger	berufstätige	reichen	geringes_einkommen
wissenschaftler	sozialen_bereich	berufstätigen	viel_geld	amt
banker	pflegeberufe	personal	wohlhabende	schlecht_bezahlte
bänker	pflegeberufen	beschäftigte	reichtum	prekäre
banken	gesundheitswesen	beschäftigten	wohlhabend	existenzminimum
geschäftsführer	rettungsdienst	mitarbeiter	millionäre	einkommen
selbstständige	mediziner	steuerzahler	erben	lohn
selbstständig	sozialarbeiter	9_to_5	geerbt	gehalt
chefs	sanitäter	gearbeitet	vermögen	geld
vorstände	handwerker	angestellt	vermögend	besitz
wirtschaftsbosse	handwerk	wirtschaft	luxus	besitzen
juristen	handwerksberufe	arbeitslose	hohes_einkommen	finanziell
anwälte	handwerkliche	arbeitslos	wohlstand	finanziellen
firmen	handwerkliche_berufe	arbeitslosigkeit	unternehmen	finanzielles
steuerberater	handwerklichen_berufen	nicht_arbeiten	superreiche	steuern
management	arbeiter	keine_arbeit	superreichen	eigentum
geschäftsleute	landwirte	ohne_arbeit	autos	verdiener
firmenchefs	facharbeiter	alg_i	großverdiener	urlaub
geschäftsmänner	meister	alg_ii	gutverdienende	reisen
professoren	bäcker	arbeitslosengeld_ii	gutes_einkommen	haus
lobbyisten	it	arbeitslosengeld	bonzen	knete
polizei	digital	rente	obere_zehntausend	auto
polizisten	internet	influencer	oberen_zehntausend	verdienende
feuerwehr	informatiker	influenzer	vielverdiener	miete
polizeibeamte	technik	sozialen_medien	finanziell_gesichert	bezahlt
soldaten	verkäufer	soziale_medien	finanziell_abgesichert	bezahlen
beamte	einzelhandel	social_media	finanziell_unabhängig	verdienst
lehrer	niedriglohnsektor	sportler	obdachlose	wirtschaftlich
öffentlichen_dienst	dienstleister	profisportler	arme	lebensunterhalt
öffentlicher_dienst	reinigungskräfte	fußballer	geringverdiener	
höheren_dienst	angestellte	fussballer	gering_verdiener	
höherer_dienst	arbeitende	profifußballer	arm	
mittlerer_dienst	arbeit	profi_sportler	armut	
mittleren_dienst	beruf	profi_fußballer	ärmer	
dienst	berufe	profi_fussballer	wenig_geld	
soziale_berufe	berufen	formel_1	empfänger	
sozialen_berufen	berufliche	promis	hartz_4	
pfleger	job	prominente	hartz4	
pflegerinnen	jobs	stars	hartz_iv	
pflegepersonal	arbeitnehmer	berühmte	harz_4	
pflegende	arbeiten	sänger	harz_iv	
pflegekräfte	normale_arbeiter	journalisten	sozial_schwache	
pflege	normalen_arbeiter		sozialhilfeempfänger	
medizinisches	fachkräfte		sozialleistungen	
medizinischen	hart_arbeitende		sparen	
krankenhäusern	hart_arbeitend		runden_kommen	
krankenhäuser	klein_unternehmer		in_den_mund	
krankenschwester	kleinunternehmer		schmarotzer	

Table B9: Socioeconomic Terms - English Translation

occupation	occupation (I)	occupation (II)	wealth	wealth (I)
doctors	nurses	employees	rich	social parasites
managers	educators	labor market	wealthy	low income
entrepreneurs	caregivers	working people	rich	low_income
scientists	social sector	workers	a lot of money	office
bankers	nursing professions	personnel	wealthy people	poorly paid
bankers	nursing occupations	employed	wealth	precarious
banks	healthcare	employees	wealthy	subsistence level
managing directors	emergency services	staff	millionaires	income
self-employed	doctors	taxpayers	heirs	wage
self-employed	social workers	9_to_5	inherited	salary
bosses	paramedics	worked	assets	money
executives	craftsmen	employed	wealthy	possession
economic bosses	trades	unemployed	luxury	own
lawyers	skilled trades	unemployment	high income	financially
attorneys	tradespeople	unemployed	prosperity	financial
companies	skilled occupations	not working	super-rich	financials
tax advisors	skilled trade professions	no work	super rich	taxes
management	workers	no job	cars	property
business people	farmers	unemployment benefits I	high earners	vacation
company heads	skilled workers	unemployment benefits II	high earners	travel
businessmen	masters	unemployment benefits II	good income	house
professors	bakers	unemployment benefits	big shots	dough
lobbyists	IT	pension	upper ten thousand	car
police	digital	influencer	upper ten thousand	earners
policemen	internet	influencers	high earners	rent
firefighters	IT specialists	social media	financially secure	paid
police officers	technology	social media	financially secure	pay
soldiers	salespeople	social media	financially independent	earnings
civil servants	retail	athletes	homeless	economically
teachers	low-wage sector	professional athletes	poor	livelihood
public service	service providers	soccer players	low earners	
public sector	cleaners	football players	low earner	
higher service	employees	professional soccer players	poor	
higher service	workers	professional athletes	poverty	
middle service	work	professional soccer players	poorer	
middle service	profession	professional footballers	little money	
service	professions	formula 1	recipients	
social professions	professions	celebrities	Hartz 4	
social professions	job	celebrities	Hartz4	
caregivers	jobs	stars	Hartz IV	
caregivers	workers	famous	Hartz 4	
nursing staff	work	singers	Hartz IV	
caregivers	normal workers	journalists	socially weak	
nursing staff	normal workers		welfare recipients	
nursing	skilled workers		social benefits	
medical	hard-working		saving	
medical staff	hard-working		making ends meet	
hospitals	small entrepreneurs		making ends meet	
hospitals	small business owners		parasites	

Table B10: Sociocultural Terms

gender	sexuality	race	marginalized
frauen	homosexuelle	migranten	minderheiten
männer	homo	migrationshintergrund	randgruppen
geschlecht	lgbtq	ausländer	rand
frau	lgbtqia	zuwanderer	
mann	lgbt	flüchtlinge	
feministen	lgbtx	kanacken	
feministinnen	schwule	einwanderer	
mütter	schwul	integration	
weisse_männer	lesben	geflüchtete	
weiße_männer	lesbisch	deutsche	
	queere	deutschen	
	queer	weiße	
	gay	deutschland	
	heterosexuelle	volk	
	transgender	einheimische	
	transsexuell	weisse	
	transsexuelle	herkunft	
	trans menschen	integrieren	
		bipoc	
		bpoc	
		poc	

Table B11: Sociocultural Terms - English Translation

gender	sexuality	race	marginalized
women	homosexuals	migrants	minorities
men	homo	migration background	fringe groups
gender	lgbtq	foreigners	fringe
woman	lgbtqia	immigrants	
man	lgbt	refugees	
feminists	lgbtx	racial slur	
feminists (f)	gays	immigrants	
mothers	gay	integration	
white men	lesbians	refugees	
white men	lesbian	Germans	
	queer	Germans	
	queer	whites	
	gay	Germany	
	heterosexual	people	
	transgender	locals	
	transsexual	whites	
	transsexuals	origin	
	trans people	integrate	
		bipoc	
		bpoc	
		poc	

Table B12: Education Terms

low	high	generic
bildungsfern	gebildete	bildung
ungelernt	gebildet	ausbildung
ungelernte	studiert	weiterbilden
ungebildet	studierte	lernen
ungebildete	studierten	bildungsgrad
schlechte_bildung	akademiker	schulbildung
keine_bildung	guter_ausbildung	bildungsniveau
mangelnde_bildung	gute_ausbildung	ausbildungsberufe
geringe_bildung	gute_bildung	ausgebildete
ohne_ausbildung	studieren	bildungsstand
	bessere bildung	qualifikation
	besserer_bildung	bildungschancen
	studenten	bildungsabschluss
	abitur	schulabschluss
	studierende	schule
	bildungsbürgertum	abschluss
	studium	

Table B13: Education Terms - English Translation

low	high	generic
educationally deprived	educated	education
unskilled	educated	training
unskilled	studied	further education
uneducated	graduates	learning
uneducated	graduates	educational level
poor education	academics	schooling
no education	good education	education level
lack of education	good education	training professions
low education	good education	trained
without education	study	educational status
	better education	qualification
	better education	educational opportunities
	students	educational attainment
	high school diploma	school graduation
	students	school
	educated middle class	degree
	degree	

Table B14: Attributes

personality	personality (I)	attributes
arrogant	sorgen	leisten
arroganz	klein	leistungen
arrogante	kleine	verdienen
egoistisch	sozial	verdient
egoisten	soziale	versuchen
schlecht	offen	nach_oben
schlechte	weltoffen	kämpfen
schlechter	zielstrebig	mut
abgehoben	fleißig	erreichen
überheblich	fleißige	mühe
egozentrisch	zufrieden	erfolg
oberflächlich	bodenständig	erfolgreich
skrupellos	engagiert	erledigen
faul	engagement	leistungsträger
faule	engagieren	bemühen
lügner	ehrgeizig	mehrwert
schwache	glücklich	glück
über_leichen	hilfsbereit	vorteil
korrupt	interessiert	chancen
gier	unabhängig	chance
betrüger	aktiv	teilen
auf_kosten	arbeitssam	kümmern
nicht_bereit	wichtig	dienen
abhängig	wichtigen	sozialen
angeber	wichtige	für_andere
normale	gemeinwohl	einsetzen
normal	systemrelevant	pech
einfach	systemrelevante	geraten
einfache	freundlich	wissen
einfachen	lieb	mittelschicht
normalen	ehrlich	mittelstand
wie_ich		mitte
neues		mittleren
neue		mittelständler
neu		mittelklasse
alleine		
unauffällig		
durchschnitt		
durchschnittlichem		
durchschnittliches		

Table B15: Attributes - English Translation

personality	personality (I)	attributes
arrogant	worries	achieve
arrogance	small	achievements
arrogant (f)	small (f)	earn
selfish	social	earned
egoists	social (f)	try
bad	open	upward
bad (f)	cosmopolitan	fight
worse	determined	courage
aloof	diligent	achieve
conceited	diligent (f)	effort
egocentric	satisfied	success
superficial	down-to-earth	successful
ruthless	committed	accomplish
lazy	commitment	high achievers
lazy (f)	commit	effort
liars	ambitious	added value
weak	happy	luck
over bodies	helpful	advantage
corrupt	interested	opportunities
greed	independent	chance
fraudsters	active	share
at the expense	hardworking	care
not willing	important	serve
dependent	important (f)	social
show-offs	important (f)	for others
normal	common good	engage
normal	system relevant	bad luck
simple	system relevant (f)	get into
simple (f)	friendly	know
simple (pl)	kind	middle class
normal (pl)	honest	middle class
like me		middle
new		middle
new (f)		middle class
new (n)		middle class
alone		
inconspicuous		
average		
average (adj)		
average (n)		

Table B16: Political Terms

power	groups	environment
politik	querdenker	umwelt
macht	konservativ	umweltaktivisten
politiker	konservative	umweltschützer
parteien	rechtsradikale	veganer
regierung	nazis	klimaaktivisten
politisch	schwurbler	
politische	grüne	
politischen	grünen	
lobbyisten	aktivisten	
lobby	rechte	
	weltverbesserer	
	linke	

Table B17: Political Terms - English Translation

power	groups	environment
politics	lateral thinkers	environment
power	conservative	environmental activists
politicians	conservatives	environmentalists
parties	right-wing radicals	vegans
government	Nazis	climate activists
political	conspiracy theorists	
political (adj)	Greens	
political (pl)	Greens (pl)	
lobbyists	activists	
lobby	right-wing	
	do-gooders	
	left-wing	

Table B18: No Content Terms

weiß_nicht
weis_nicht
weiss_nicht
weiss_ich_nicht
weiß_ich_nicht
weis_ich_nicht
weiß_es_nicht
keine_ahnung
keine_richtige_ahnung
nicht_die_geringste_ahnung
kene_ahnung
kann_ich_nicht_sagen
fällt_dazu_nichts
fällt_mir
fällt_nicht
fallt_nichts
fällt_nix
keine_angabe
keine_angaben
ka
k.a

Table B19: No Content Terms - English Translation

don’t know
don’t know
don’t know
I don’t know
I don’t know
I don’t know
I don’t know it
no idea
no real idea
not the slightest idea
no idea
can’t say
can’t think of anything
comes to mind
don’t think
can’t think
can’t think of anything
no comment
no comments
idk
n/a

C. Additional results and regression tables

C1. Results for medium status group (static)

Figure C1: Mid status groups - absolute frequencies, pooled sample

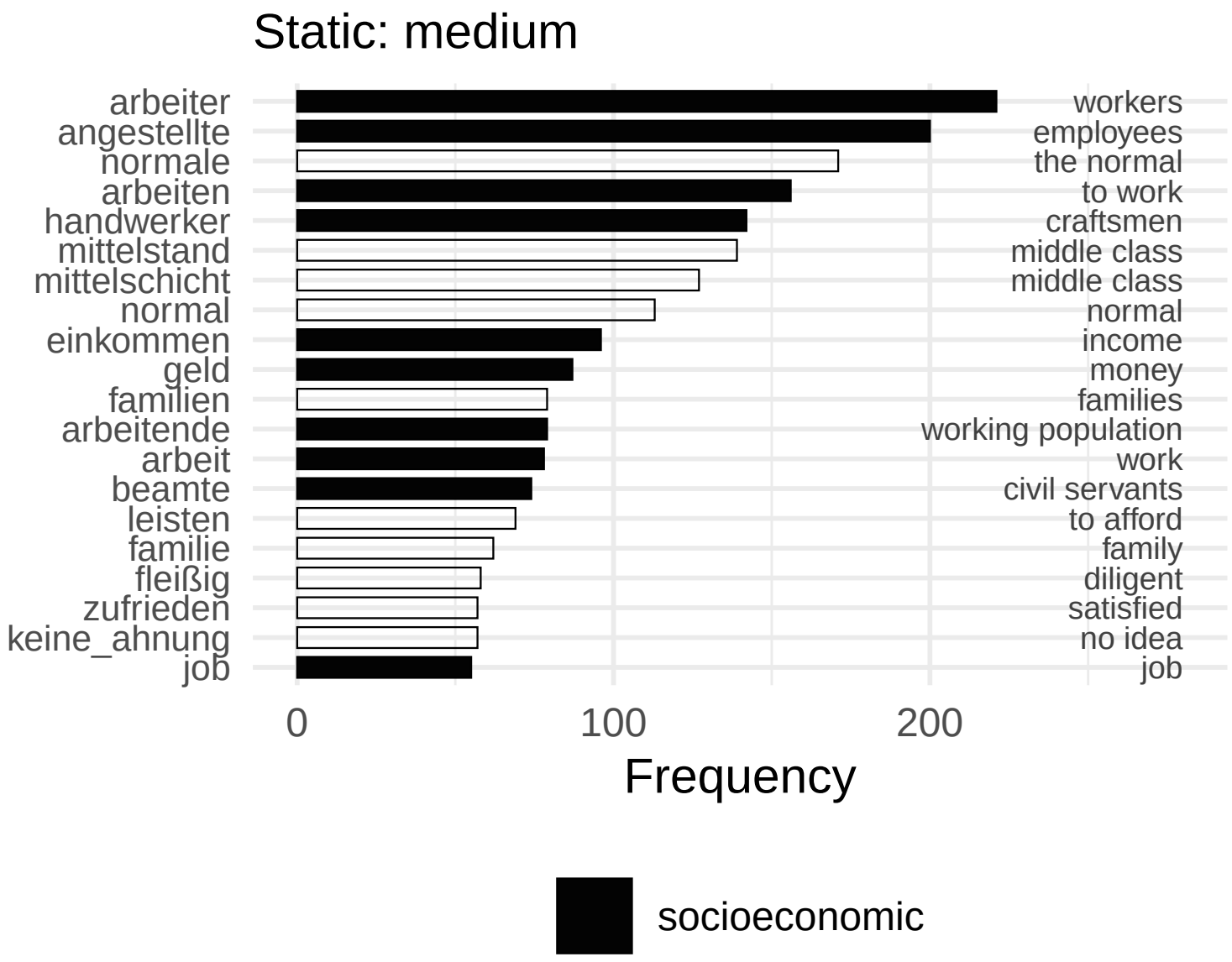

C2. Salience of dictionary (sub)dimensions

Table C1: Salience of all dictionary dimensions by question type

Dimension	Question	N	%
Socioeconomic	static	5183	47.2
	dynamic	2820	38.4
Sociocultural	static	529	4.8
	dynamic	847	11.5
Education	static	940	8.6
	dynamic	647	8.8
Politics	static	787	7.2
	dynamic	759	10.3
Attributes	static	2820	25.7
	dynamic	1379	18.8
Dontknow	static	352	3.2
	dynamic	322	4.4
Total	static	10987	93.4
	dynamic	7349	87.8

Note: Table displays the percentage of answers mentioning the dimension per question type. The dynamic category includes the two questions about winning and losing groups, the static category the three questions about upper, medium and low groups. The 'Total' rows show the number of answers per answer category and the percentage of answers that contain any of the dictionary terms. Thus, 93% of the answers to the static questions contain any of the five listed dictionary dimensions, while 87% of the dynamic questions do (excluding the 'don't know' dimension).

Table C2: Salience of dictionary subdimensions by question type

Question	Dimension	Subdimension	N	pct
static	Socioeconomic	Occupation	2996	27.3
		Wealth	3005	27.4
	Sociocultural	Gender	73	0.7
		Sexuality	14	0.1
		Race	434	4.0
	Education	Education	940	8.6
	Total	Total	10987	100.0
dynamic	Socioeconomic	Occupation	2221	30.2
		Wealth	868	11.8
	Sociocultural	Gender	390	5.3
		Sexuality	175	2.4
		Race	406	5.5
	Education	Education	647	8.8
	Total	Total	7349	100.0

Note: Table displays the percentage of answers mentioning the dimension. The dynamic category includes the two questions about winning and losing groups, the static category the three questions about upper, medium and low groups. The 'Total' rows show the number of answers per question type.

C3. Individual-level analysis of dictionary mentions

Figure C2: Predicted probabilities of mentioning socioeconomic groups as winning or losing, by attitudinal subgroup

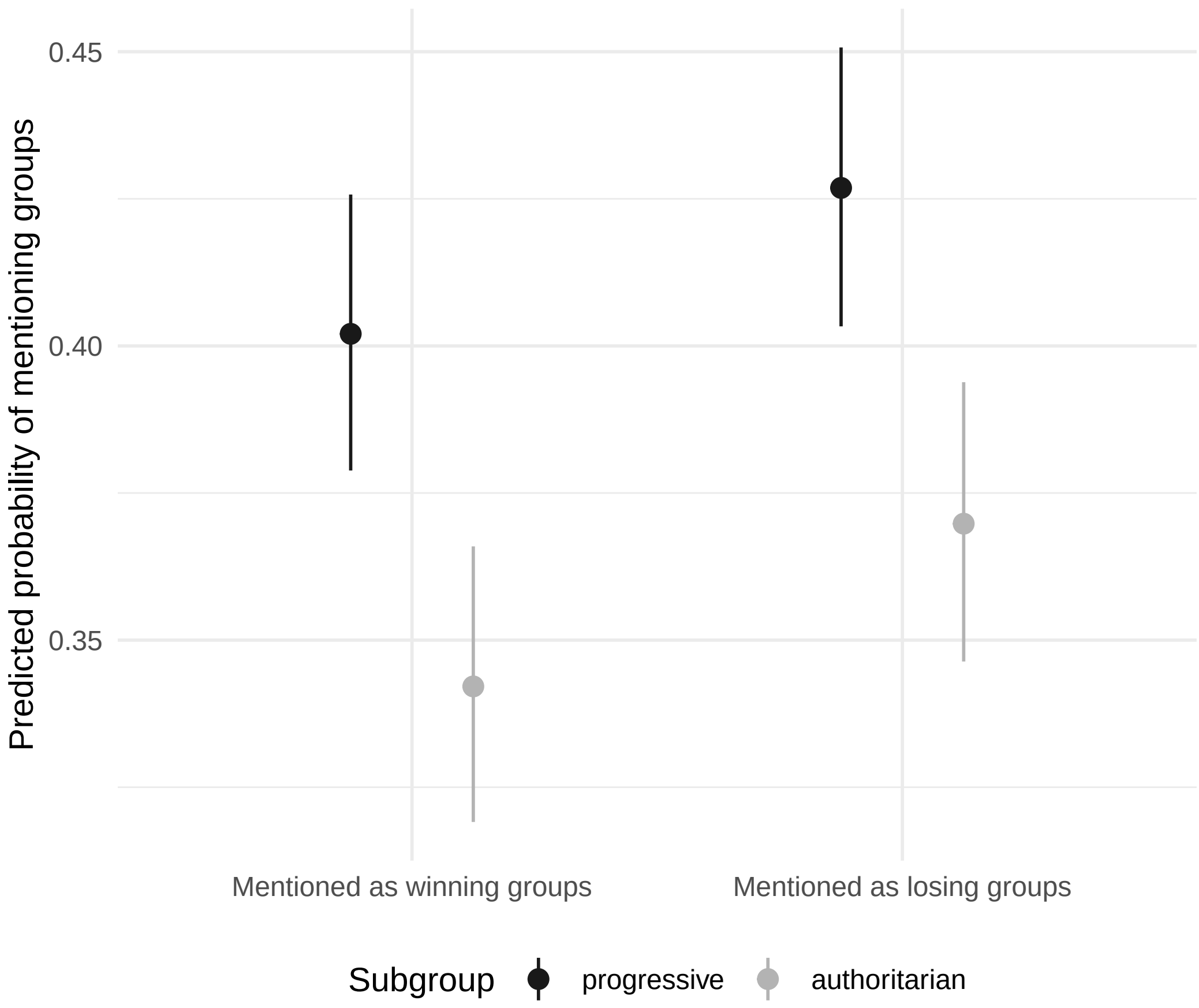

Marginal predictions of a binomial generalized linear model, controlling for redistribution attitude, gender, age, education, income, urban/rural residence and migration background. Confidence intervals: 95%, see table C7 for full models.

Figure C3: Predicted probabilities of mentioning sociocultural groups as winning or losing, by GAL-TAN attitudes (continuous, polynomial term)

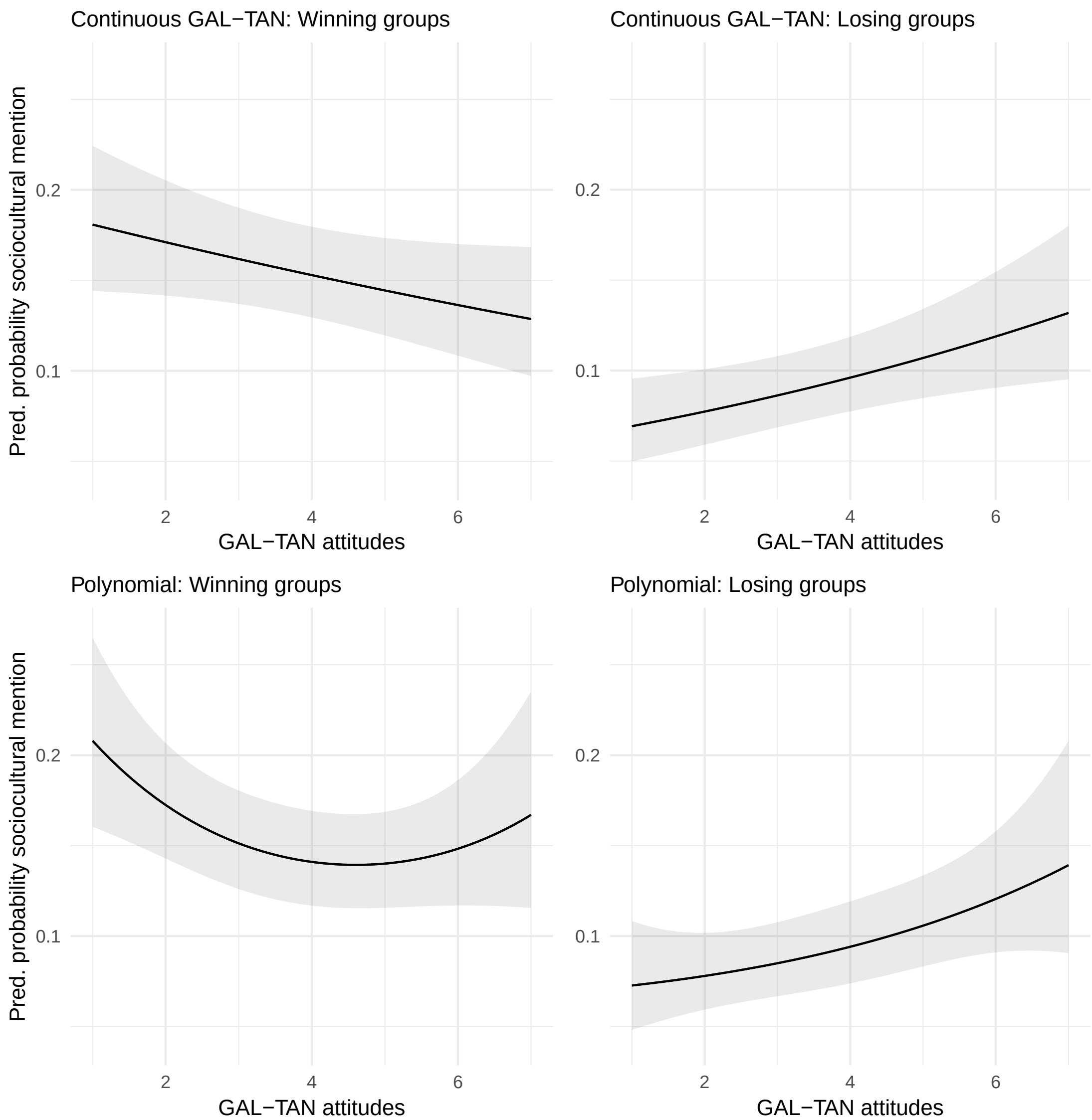

Conditional predictions of a binomial generalized linear model, controlling for redistribution attitude, gender, age, education, income, urban/rural residence and migration background. Confidence intervals: 95%, see table C3 for full models.

Figure C4: Predicted probabilities of mentioning sociocultural groups as winning or losing, by migration background (yes = 543, no = 3264 respondents)

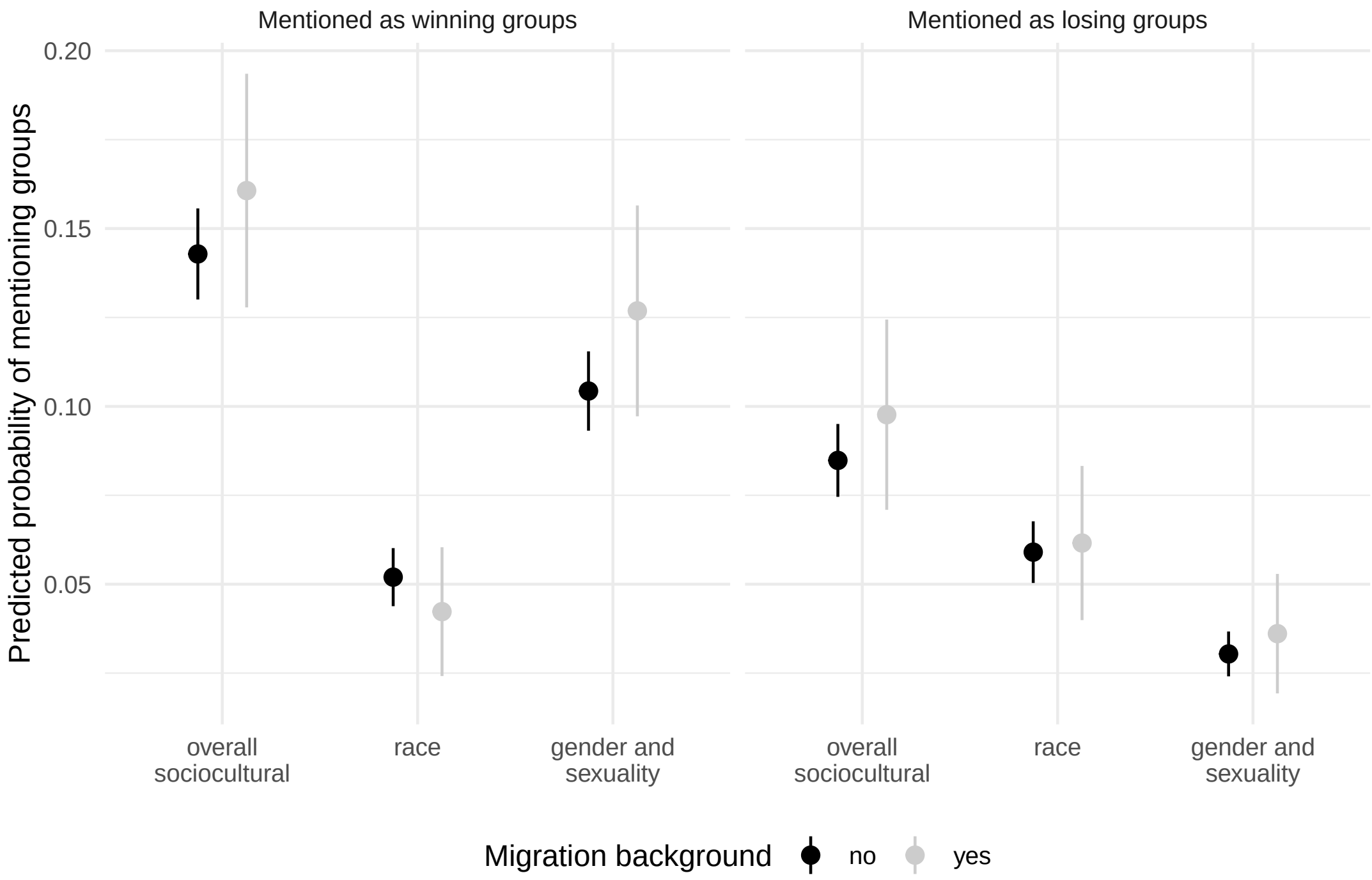

Marginal predictions of a binomial generalized linear model, controlling for sociocultural attitudes, redistribution attitude, gender, age, education, income, and urban/rural residence. Confidence intervals: 95%.

Figure C5: Predicted probabilities of mentioning sociocultural groups as winning or losing, by gender and age

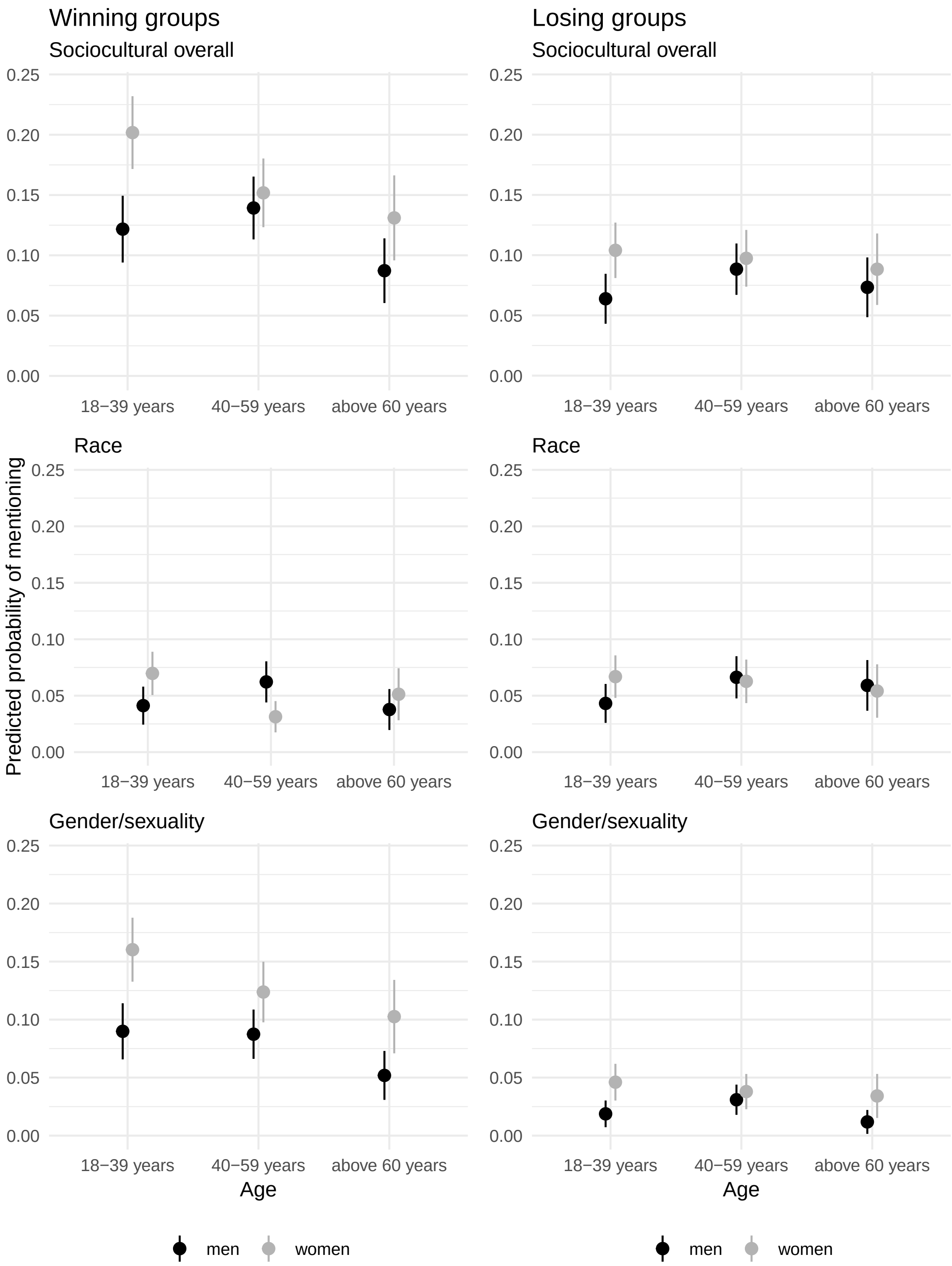

Marginal predictions of a binomial generalized linear model, controlling for sociocultural attitudes, redistribution attitude, education, income, urban/rural residence and migration background. Confidence intervals: 95%.

Figure C6: Keyness scores - Subgroup comparison of groups mentioned as winning status by young men (bottom) vs. young women (top)

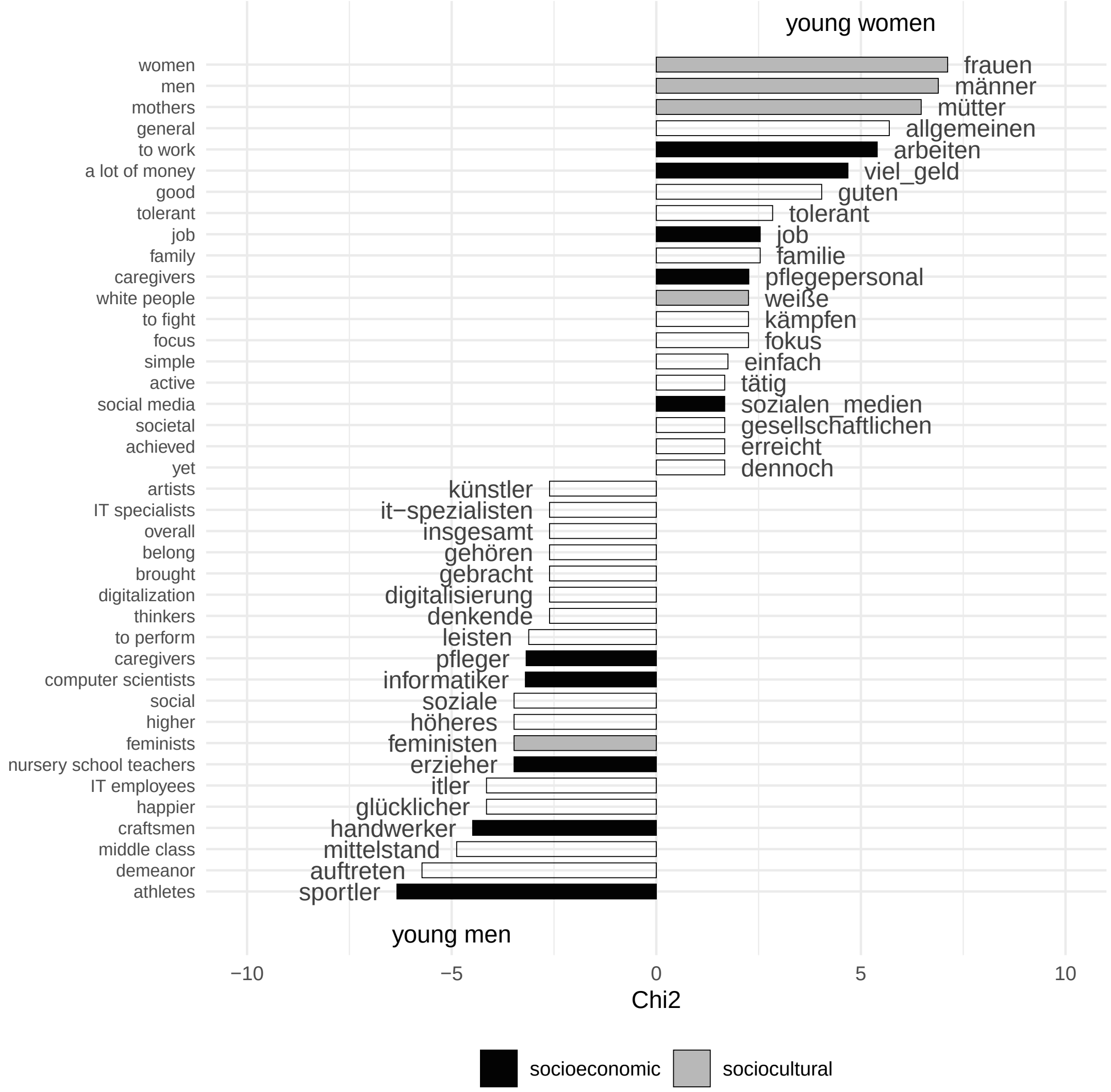

The plot displays the 20 most characteristic words used to describe losing groups, by progressive (top) vs. authoritarian attitudes (bottom). The more extreme the Chi-squared statistic (= keyness score) on the x-axis is, the more characteristic (or comparatively frequent) a term is for one group compared to the other.

Table C3: Predictors of mentioning sociocultural dictionary dimension (binary), GAL-TAN attitude specifications and all controls

	Winning	Winning 2	Winning 3	Losing	Losing 2	Losing 3
Social conservative group	−0.170			0.226		
	(0.115)			(0.142)		
TAN attitudes (cont.)		−0.067+	−0.343*		0.119**	0.049
		(0.038)	(0.143)		(0.046)	(0.187)
TAN attitudes^2			0.037*			0.009
			(0.019)			(0.023)
Anti-redistribution attitude (cont.)	−0.048	−0.038	−0.029	0.007	−0.017	−0.015
	(0.031)	(0.033)	(0.033)	(0.038)	(0.040)	(0.040)
Age	−0.012***	−0.011***	−0.011**	0.000	−0.001	−0.001
	(0.003)	(0.003)	(0.003)	(0.004)	(0.004)	(0.004)
Woman	0.411***	0.407***	0.401***	0.275*	0.280*	0.278*
	(0.103)	(0.103)	(0.103)	(0.128)	(0.128)	(0.128)
Medium education (ref. low)	−0.050	−0.055	−0.052	0.035	0.049	0.050
	(0.137)	(0.137)	(0.137)	(0.167)	(0.167)	(0.167)
High education (ref. low)	0.111	0.099	0.097	−0.034	0.001	0.000
	(0.157)	(0.157)	(0.157)	(0.199)	(0.199)	(0.199)
Household-equivalized income	0.000	0.000	0.000	0.000	0.000	0.000
	(0.000)	(0.000)	(0.000)	(0.000)	(0.000)	(0.000)
With migration background	0.019	0.021	0.025	0.115	0.110	0.111
	(0.139)	(0.139)	(0.140)	(0.172)	(0.173)	(0.173)
Suburbs/small city (ref. country)	0.019	0.016	0.017	0.125	0.131	0.132
	(0.128)	(0.128)	(0.128)	(0.164)	(0.164)	(0.164)
Big city (ref. country)	0.129	0.130	0.129	0.391*	0.393*	0.393*
	(0.142)	(0.142)	(0.142)	(0.179)	(0.179)	(0.179)
Num.Obs.	3301	3301	3301	3301	3301	3301
AIC	2711.1	2710.1	2708.2	1953.3	1949.3	1951.1
BIC	2778.2	2777.2	2781.4	2020.4	2016.4	2024.3
Log.Lik.	−1344.534	−1344.047	−1342.098	−965.664	−963.635	−963.561
F	4.782	4.892	4.857	1.425	1.826	1.685
RMSE	0.35	0.35	0.35	0.28	0.28	0.28

+ p <0.1, * p <0.05, ** p <0.01, *** p <0.001
Binomial generalized linear model. Standard errors in parentheses, conf. level: 0.95.

Table C4: Predictors of mentioning racial dictionary dimension (binary), GAL-TAN attitude specifications and all controls

	Winning	Winning 2	Winning 3	Losing	Losing 1	Losing 3
Social conservative group	0.074			0.241		
	(0.183)			(0.169)		
TAN attitudes (cont.)		0.006	−0.290		0.126*	0.034
		(0.060)	(0.229)		(0.055)	(0.223)
TAN attitudesˆ2			0.039			0.012
			(0.029)			(0.028)
Anti-redistribution attitude (cont.)	−0.005	0.001	0.011	0.024	−0.002	0.001
	(0.050)	(0.052)	(0.052)	(0.046)	(0.047)	(0.047)
Age	−0.010+	−0.010+	−0.010+	0.001	0.001	0.001
	(0.005)	(0.005)	(0.005)	(0.005)	(0.005)	(0.005)
Woman	0.059	0.059	0.051	0.108	0.114	0.112
	(0.163)	(0.163)	(0.163)	(0.151)	(0.151)	(0.152)
Medium education (ref. low)	0.061	0.056	0.058	−0.044	−0.030	−0.029
	(0.219)	(0.219)	(0.219)	(0.195)	(0.195)	(0.195)
High education (ref. low)	0.259	0.247	0.242	−0.071	−0.035	−0.037
	(0.250)	(0.251)	(0.251)	(0.233)	(0.233)	(0.233)
Household-equivalized income	0.000	0.000	0.000	0.000	0.000	0.000
	(0.000)	(0.000)	(0.000)	(0.000)	(0.000)	(0.000)
With migration background	−0.348	−0.347	−0.343	0.025	0.020	0.021
	(0.247)	(0.247)	(0.247)	(0.211)	(0.211)	(0.211)
Suburbs/small city (ref. country)	0.307	0.306	0.306	−0.043	−0.036	−0.036
	(0.222)	(0.222)	(0.222)	(0.192)	(0.192)	(0.192)
Big city (ref. country)	0.532*	0.531*	0.531*	0.335	0.337	0.337
	(0.238)	(0.238)	(0.238)	(0.207)	(0.207)	(0.207)
Num.Obs.	3301	3301	3301	3301	3301	3301
AIC	1330.5	1330.7	1331.0	1499.8	1496.7	1498.5
BIC	1397.7	1397.8	1404.2	1567.0	1563.8	1571.7
Log.Lik.	−654.269	−654.346	−653.477	−738.922	−737.349	−737.260
F	1.335	1.322	1.362	0.935	1.245	1.163
RMSE	0.22	0.22	0.22	0.24	0.24	0.24

+ p <0.1, * p <0.05, ** p <0.01, *** p <0.001
Binomial generalized linear model. Standard errors in parentheses, conf. level: 0.95.

Table C5: Predictors of mentioning gender and/or sexuality dictionary dimensions (binary), GAL-TAN attitude specifications and all controls

	Winning	Winning 2	Winning 3	Losing	Losing 2	Losing 3
Social conservative group	−0.217+			0.301		
	(0.132)			(0.231)		
TAN attitudes (cont.)		−0.081+	−0.277+		0.162*	0.067
		(0.043)	(0.164)		(0.073)	(0.297)
TAN attitudes^2			0.027			0.012
			(0.022)			(0.037)
Anti-redistribution attitude (cont.)	−0.082*	−0.072+	−0.065+	−0.089	−0.123+	−0.120+
	(0.036)	(0.038)	(0.038)	(0.063)	(0.064)	(0.065)
Age	−0.015***	−0.014***	−0.014***	−0.007	−0.008	−0.008
	(0.004)	(0.004)	(0.004)	(0.007)	(0.007)	(0.007)
Woman	0.602***	0.597***	0.593***	0.620**	0.628**	0.625**
	(0.120)	(0.120)	(0.120)	(0.214)	(0.214)	(0.214)
Medium education (ref. low)	−0.059	−0.065	−0.062	0.237	0.259	0.260
	(0.157)	(0.157)	(0.157)	(0.291)	(0.291)	(0.291)
High education (ref. low)	0.016	0.003	0.002	0.239	0.296	0.294
	(0.181)	(0.181)	(0.181)	(0.335)	(0.336)	(0.336)
Household-equivalized income	0.000*	0.000*	0.000*	0.000	0.000	0.000
	(0.000)	(0.000)	(0.000)	(0.000)	(0.000)	(0.000)
With migration background	0.095	0.096	0.099	0.064	0.059	0.061
	(0.155)	(0.155)	(0.155)	(0.275)	(0.275)	(0.275)
Suburbs/small city (ref. country)	−0.040	−0.042	−0.041	0.552+	0.561+	0.562+
	(0.143)	(0.143)	(0.143)	(0.289)	(0.289)	(0.289)
Big city (ref. country)	−0.050	−0.049	−0.049	0.564+	0.567+	0.567+
	(0.162)	(0.162)	(0.162)	(0.317)	(0.317)	(0.317)
Num.Obs.	3301	3301	3301	3301	3301	3301
AIC	2207.6	2206.8	2207.3	920.5	917.4	919.3
BIC	2274.7	2273.9	2280.5	987.7	984.6	992.6
Log.Lik.	−1092.786	−1092.398	−1091.638	−449.268	−447.720	−447.667
F	6.578	6.680	6.275	1.754	2.064	1.889
RMSE	0.31	0.31	0.31	0.17	0.17	0.17

+ p <0.1, * p <0.05, ** p <0.01, *** p <0.001
Binomial generalized linear model. Standard errors in parentheses, conf. level: 0.95.

Table C6: Predictors of mentioning sociocultural dictionary dimensions (binary), by gender and education

	Winning			Losing		
	soc-cult	race	gender	soc-cult	race	gender
Women without higher ed.	−0.168	−0.424+	−0.076	0.010	−0.161	−0.004
	(0.157)	(0.254)	(0.173)	(0.212)	(0.252)	(0.315)
Men with higher ed.	−0.441*	−0.366	−0.629**	−0.323	−0.355	−0.499
	(0.185)	(0.294)	(0.214)	(0.249)	(0.293)	(0.384)
Men without higher ed.	−0.554***	−0.359	−0.652***	−0.260	−0.189	−0.713*
	(0.167)	(0.257)	(0.189)	(0.220)	(0.256)	(0.351)
TAN attitudes (cont.)	−0.065+	0.009	−0.078+	0.118*	0.130*	0.154*
	(0.038)	(0.060)	(0.043)	(0.046)	(0.055)	(0.073)
Anti-redistribution attitude (cont.)	−0.038	0.002	−0.072+	−0.017	−0.001	−0.123+
	(0.033)	(0.052)	(0.038)	(0.040)	(0.047)	(0.064)
Age	−0.011***	−0.010+	−0.014***	0.000	0.001	−0.008
	(0.003)	(0.005)	(0.004)	(0.004)	(0.005)	(0.007)
Household-equivalized income	0.000	0.000	0.000*	0.000	0.000	0.000
	(0.000)	(0.000)	(0.000)	(0.000)	(0.000)	(0.000)
With migration background	0.021	−0.341	0.096	0.111	0.021	0.063
	(0.139)	(0.247)	(0.155)	(0.173)	(0.211)	(0.275)
Suburbs/small city (ref. country)	0.017	0.297	−0.041	0.129	−0.039	0.557+
	(0.128)	(0.222)	(0.143)	(0.164)	(0.192)	(0.289)
Big city (ref. country)	0.130	0.523*	−0.048	0.391*	0.335	0.565+
	(0.142)	(0.238)	(0.162)	(0.179)	(0.207)	(0.317)
Num.Obs.	3301	3301	3301	3301	3301	3301
AIC	2710.2	1329.2	2206.9	1949.3	1495.8	918.1
BIC	2777.3	1396.4	2274.0	2016.4	1562.9	985.2
Log.Lik.	−1344.098	−653.621	−1092.461	−963.662	−736.899	−448.031
F	4.900	1.493	6.684	1.819	1.332	1.995
RMSE	0.35	0.22	0.31	0.28	0.24	0.17

+ p <0.1, * p <0.05, ** p <0.01, *** p <0.001
Binomial generalized linear model (reference category: women with higher education). Standard errors in parentheses, conf. level: 0.95.

Table C7: Predictors of mentioning socioeconomic dictionary dimensions (binary), GAL-TAN attitude specifications and all controls

	Winning	Winning 2	Winning 3	Losing	Losing 2	Losing 3
Social conservative group	−0.204*			−0.208*		
	(0.083)			(0.082)		
TAN attitudes (cont.)		−0.086**	−0.007		−0.063*	−0.036
		(0.027)	(0.109)		(0.027)	(0.107)
TAN attitudesˆ2			−0.010			−0.004
			(0.014)			(0.014)
Anti-redistribution attitude (cont.)	0.008	0.022	0.020	−0.006	−0.003	−0.003
	(0.023)	(0.024)	(0.024)	(0.022)	(0.023)	(0.024)
Age	0.001	0.001	0.001	0.003	0.004	0.004
	(0.002)	(0.002)	(0.002)	(0.002)	(0.002)	(0.002)
Woman	0.212**	0.208**	0.209**	0.171*	0.168*	0.169*
	(0.074)	(0.074)	(0.074)	(0.073)	(0.073)	(0.073)
Medium education (ref. low)	0.407***	0.400***	0.399***	0.109	0.109	0.109
	(0.101)	(0.101)	(0.101)	(0.097)	(0.097)	(0.097)
High education (ref. low)	0.458***	0.442***	0.443***	0.213+	0.214+	0.214+
	(0.118)	(0.118)	(0.118)	(0.114)	(0.114)	(0.114)
Household-equivalized income	0.000*	0.000+	0.000+	0.000+	0.000+	0.000+
	(0.000)	(0.000)	(0.000)	(0.000)	(0.000)	(0.000)
With migration background	−0.191+	−0.188+	−0.190+	−0.315**	−0.314**	−0.314**
	(0.107)	(0.107)	(0.107)	(0.107)	(0.107)	(0.107)
Suburbs/small city (ref. country)	−0.028	−0.032	−0.032	0.041	0.039	0.039
	(0.091)	(0.091)	(0.091)	(0.089)	(0.089)	(0.089)
Big city (ref. country)	−0.159	−0.159	−0.159	−0.289**	−0.289**	−0.289**
	(0.104)	(0.105)	(0.105)	(0.104)	(0.104)	(0.104)
Num.Obs.	3301	3301	3301	3301	3301	3301
AIC	4333.9	4330.1	4331.5	4414.5	4415.5	4417.4
BIC	4401.0	4397.2	4404.8	4481.6	4482.6	4490.6
Log.Lik.	−2155.934	−2154.041	−2153.764	−2196.226	−2196.728	−2196.695
F	5.381	5.742	5.257	5.057	4.958	4.512
RMSE	0.48	0.48	0.48	0.49	0.49	0.49

+ $p < 0.1$, * $p < 0.05$, ** $p < 0.01$, *** $p < 0.001$
Binomial generalized linear model. Standard errors in parentheses, conf. level: 0.95.

C4. Tests of zero-sum thinking

Table C8: More narrow dictionary dimensions to test zero-sum thinking

Category	Dictionary terms German	Dictionary terms translated
Migrants/PoC	*migrant*, migrationshintergrund*, ausländ*, zuwander*, flüchtling*, kanacke*, einwander*, geflüchtet*, asylant*, fremd* herkunft*, fliehen, geflohen*, bipoc, bpoc, poc	*migrant*, migration background*, foreign*, immigrant*, refugee*, Kanacke* [racial slur], immigrant*, fled*, asylum seeker*, foreign* origin*, flee, fled*, bipoc, bpoc, poc
Natives	deutsch*, weiße, weisse, deutschland, volk*, einheimische*, bevölkerung*, land	German*, whites, Germany, volk*, locals*/natives*, population*, country
Women	*frauen*, frau, feministen, feministinnen	*women*, woman, feminists
Men	*männer, mann	*men, man

These are dictionary dimensions on which the regressions in Table C9 are based. For example, the dependent variable in Model 1 is whether a respondent mentioned any of the terms describing natives or Germans.

Figure C7: Predicted probabilities of mentioning natives/men as losers (binary) and migrants/women as winners (binary), interacted with GAL-TAN attitudes

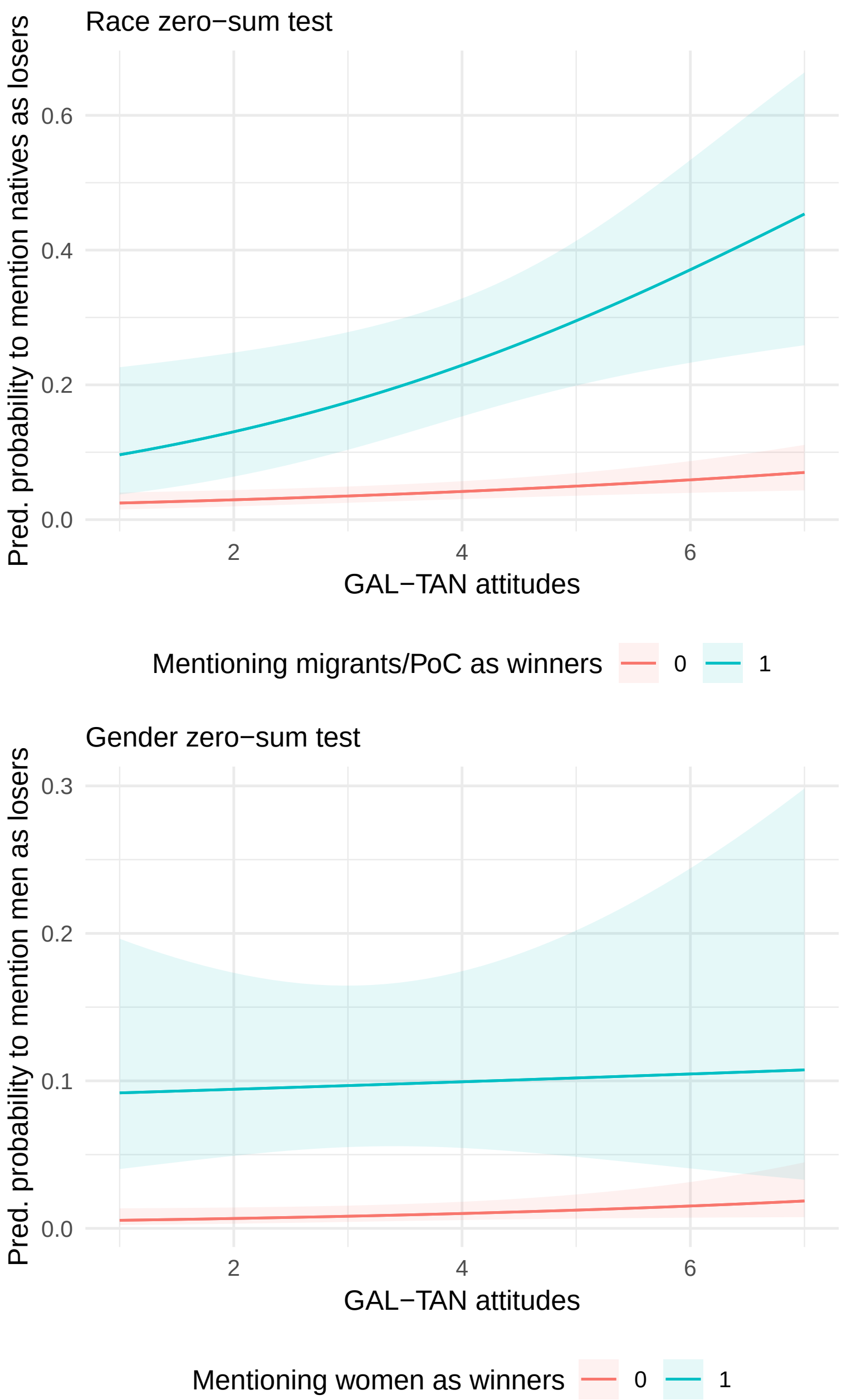

Conditional predictions of a binomial generalized linear model, controlling for redistribution attitude, gender, age, education, income, urban/rural residence and migration background. Confidence intervals: 95%, see table C9 for full models.

Table C9: Predictors of mentioning natives/men as losers (binary), by mentioning migrants/women as winners (binary) and GAL-TAN attitudes

	Natives losing 1	Natives losing 2	Men losing 1	Men losing 2
Mentioned migrants as winners	1.951***	1.278*		
	(0.223)	(0.651)		
Migrant winners x TAN att.		0.160		
		(0.143)		
Mentioned women as winners			2.478***	3.093***
			(0.284)	(0.666)
Women winners x TAN att.				−0.177
				(0.174)
TAN attitudes (cont.)	0.208***	0.182**	0.139	0.206+
	(0.061)	(0.065)	(0.099)	(0.119)
Anti-redistribution attitude (cont.)	0.000	−0.001	−0.133	−0.130
	(0.052)	(0.052)	(0.088)	(0.088)
Age	0.010+	0.010+	−0.008	−0.008
	(0.006)	(0.006)	(0.009)	(0.009)
Woman	0.032	0.024	−0.432	−0.445
	(0.169)	(0.169)	(0.278)	(0.279)
Medium education (ref. low)	0.437+	0.444+	0.497	0.475
	(0.241)	(0.242)	(0.406)	(0.406)
High education (ref. low)	0.480+	0.488+	0.383	0.352
	(0.279)	(0.279)	(0.466)	(0.465)
Household-equivalized income	0.000	0.000	0.000	0.000
	(0.000)	(0.000)	(0.000)	(0.000)
With migration background	−0.240	−0.235	−0.497	−0.477
	(0.263)	(0.263)	(0.448)	(0.447)
Suburbs/small city (ref. country)	−0.005	−0.015	0.022	0.034
	(0.214)	(0.215)	(0.347)	(0.348)
Big city (ref. country)	0.330	0.322	0.198	0.205
	(0.232)	(0.233)	(0.382)	(0.382)
Num.Obs.	3287	3287	3287	3287
AIC	1233.2	1233.9	551.4	552.4
BIC	1306.4	1313.2	624.6	631.6
Log.Lik.	−604.616	−603.969	−263.707	−263.180
RMSE	0.21	0.21	0.13	0.13

+ p <0.1, * p <0.05, ** p <0.01, *** p <0.001
Binomial generalized linear model. Standard errors in parentheses, conf. level: 0.95.
